# The neural coding framework for learning generative models

**DOI:** 10.1038/s41467-022-29632-7

**Published:** 2022-04-19

**Authors:** Alexander Ororbia, Daniel Kifer

**Affiliations:** 1 Department of Computer Science, Rochester Institute of Technology, Rochester, NY 14623 USA; 2 Department of Computer Science & Engineering, The Pennsylvania State University, State College, PA 16801 USA

**Keywords:** Dynamical systems, Learning algorithms, Network models, Computer science, Statistics

## Abstract

Neural generative models can be used to learn complex probability distributions from data, to sample from them, and to produce probability density estimates. We propose a computational framework for developing neural generative models inspired by the theory of predictive processing in the brain. According to predictive processing theory, the neurons in the brain form a hierarchy in which neurons in one level form expectations about sensory inputs from another level. These neurons update their local models based on differences between their expectations and the observed signals. In a similar way, artificial neurons in our generative models predict what neighboring neurons will do, and adjust their parameters based on how well the predictions matched reality. In this work, we show that the neural generative models learned within our framework perform well in practice across several benchmark datasets and metrics and either remain competitive with or significantly outperform other generative models with similar functionality (such as the variational auto-encoder).

## Abstract (web summary)

Brain-inspired neural generative models can be designed to learn complex probability distributions from data. Here the authors propose a neural generative computational framework, inspired by the theory of predictive processing in the brain, that facilitates parallel computing for complex tasks.

## Introduction

One way to understand how the brain adapts to its environment is to view it as a type of generative pattern-creation model^1^, one that is engaged in a never-ending process of self-correction, often without external teaching signals (or labels)^2^. Under this perspective, the brain is continuously making predictions about elements of its environment, a process that allows it to infer useful representations of the sensory data that it receives^3^ as well as to synthesize novel patterns, which could serve as the potential basis for long-term planning and imagination itself^4^. From the theoretical viewpoint of predictive processing^4^, the brain could be likened to a hierarchical model^5^ whose levels are implemented by neurons (or clusters of neurons). If levels are likened to regions of the brain, the neurons at one level (or region) attempt to predict the state of neurons at another level (or region) and adjust their local model synaptic parameters based on how different their predictions were from the observed signal. Furthermore, these neurons utilize various mechanisms to laterally stimulate and suppress each other^6^ to facilitate contextual processing (such as grouping and segmenting visual components of objects in a scene). As we will demonstrate in this article, this viewpoint can be turned into a powerful framework for learning generative models.

In machine learning, one central goal is to construct agents that learn representations that extract the underlying structure of data, without the use of explicit supervisory signals such as human-crafted labels, i.e., unsupervised learning. Generative models, or models capable of synthesizing instances of data that resemble a database of collected patterns, that are based on deep artificial neural networks (ANNs), e.g., variational autoencoders^7^ or generative adversarial networks^8^, have been shown to be one way of acquiring these representations. Once trained, an ANN model is used to fantasize patterns by injecting it with noise and propagating this noise through the system until the output nodes are reached.

However, despite the success in deploying ANNs as generative models across various applications, the way that ANNs operate and learn is a far-cry from the neuro-mechanistic story we described earlier^9,10^. Specifically, ANN generative models are trained with the popular algorithm known as back-propagation of errors (backprop)^11^, which is an elegant mathematical solution to the credit assignment problem in deep networks—synaptic weights are adjusted through the use of teaching signals that are created by propagating an error, which exists exclusively at the output of the ANN, backwards along a feedback pathway^12^, a path created by re-using the very same weights that transmitted those signals forward^13^. By virtue of this formulation, backprop imposes the constraint that the ANN takes the form of a directed feedforward structure (and does not permit the use of non-differentiable activation functions and makes integrating mechanisms such as lateral connectivity difficult). While the neurons in an ANN are usually arranged hierarchically, they do not make local predictions and they do not laterally affect each other’s activity. Furthermore, synaptic adjustment in backprop-based models is done non-locally, while in neurobiological networks this adjustment is often argued to be done locally^14–17^ (there are far more local connections than long-range connections^18^ with the neocortex adhering to a local connectivity pattern^10^). In other words, neurons make use of the information immediately available to them, in both time and space, and do not wait on distant regions in order to adjust their synapses, with global information provided through neurotransmitters such as dopamine. In response to the above problems, the statistical learning community has developed a plethora of mechanisms that modify backprop through a heuristic or additional constraint^19–22^ or, recently, has worked on developing learning procedures that embody elements of biological neuronal function while enabling backprop-level learning^12,23–27^ (see Supplementary Note 2 for a more detailed review). However, while insights from each development have proven valuable in bridging backprop with brain-like computation, many of these ideas only address one or a few of the issues described earlier and tend to focus on simple problems in classification. While the question as to how credit assignment is exactly implemented in the brain is an open one, it would prove useful to machine learning, (computational) neuroscience, and cognitive science to have a framework that demonstrates how a neural system can learn something as complex as a generative model without backprop, using mechanisms and rules that are brain-inspired.

In this work, inspired by predictive processing theory^4,28–30^ and building on free energy minimization^5,31^, which crucially casts predictive processing formally as variational Bayesian inference, we propose the neural generative coding (NGC) computational framework as a powerful way to learn generative ANNs, resolving several of the key backprop-centric issues described above. Furthermore, we show that certain settings of NGC recover the work proposed in ref. ^32^ and ref. ^5^. We find that NGC models, including ref. ^32^ and ref. ^5^, not only remain competitive with backprop-based generative ANNs across several datasets in terms of pattern creation, such as the variational autoencoder^7^, but they also outperform these models on tasks that they are not directly trained for, such as classification and pattern completion. Our results further demonstrate that, besides unifying predictive processing models, NGC allows for integration of improvements such as learnable recurrent error synapses and laterally-driven sparsity. As a result, our work presents promising evidence that brain-inspired alterations to traditional deep learning techniques can be a viable source of performance gains.

## Results

### Notation

In this paper, ⊙ indicates a Hadamard product, ⊘ indicates element-wise division, and ⋅ indicates a matrix/vector multiplication (or dot product if the two objects it is applied to are vectors of the same shape) and (**v**)^*T*^ denotes the transpose. We denote **v**_*i*_ (bold font indicates vector/matrix) means that we retrieve the *i*th element *v*_*i*_ (italic indicates single scalar element) in the vector **v** and **W**_*ij*_ means that we retrieve the element *W*_*ij*_ in the *i*th row and *j*th column of matrix **W**. ← denotes the overriding of a variable.

### Problem setting

We start with a description of the problem setting—an agent must learn to approximate a probability distribution from a dataset **X** of samples. For notational reasons, this dataset is presented in column-major order, so that each column **x** represents a record (also known as an example or item). **X** has *D* rows and *S* total columns. The items are assumed independent, so that *p*(**X**) = ∏_**x**∈**X**_ *p*(**x**) and $\log p({{{{{{{\bf{X}}}}}}}})={\sum }_{{{{{{{{\bf{x}}}}}}}}\in {{{{{{{\bf{X}}}}}}}}}\log p({{{{{{{\bf{x}}}}}}}})$. We are interested in directed generative models that are capable of producing *explicit* density estimates of the data distribution, i.e., models that estimate a probability density function (PDF) over a sample space, and we will leave the examination of most *implicit* density estimators, i.e., models that do not produce explicit density estimates of the PDF but yield a function that produces samples from the estimated distribution, based on generative adversarial networks^8^ for future work.

### The typical deep learning approach

**Fig. 1:**
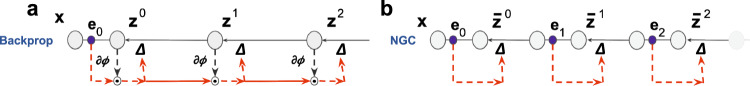
Backprop in contrast with neural coding. **a** Credit assignment in backprop requires a strict, global feedback pathway, which requires the completion of the forward pass that carries information upstream (right to left). This feedback pathway carries the error message **e**^0^ at the output layer back along (left to right) the same synapses used in the forward pass to update downstream neurons ${\bar{{{{{{{{\bf{z}}}}}}}}}}^{1}$ and ${\bar{{{{{{{{\bf{z}}}}}}}}}}^{2}$. **b** Our proposed neural generative coding (NGC) model sidesteps this neurobiologically implausible requirement by learning with short, local error transmission pathways made possible through recurrent error synapses and stateful neural activities. Credit assignment under NGC operates with local mismatch signals, **e**^1^ and **e**^2^, that readily communicate this information to their respective layers, ${\bar{{{{{{{{\bf{z}}}}}}}}}}^{1}$ and ${\bar{{{{{{{{\bf{z}}}}}}}}}}^{2}$. Black arrows indicate forward propagation while red arrows indicate backwards transmission. Solid lines indicate that a signal is transformed along a synapse(s) while dashed arrows indicate direct copying of information (in other words, it represents the identity function, or that no transformation is applied to the incoming information). ∂*ϕ* shows communication of the neuron’s first derivative, Δ represents the computed change to the synapse (of the forward pass) that will use the (nearby) error signal, and ⊙ indicates multiplication of the incoming signals.

In modern-day deep learning practice, a feedforward ANN, also called a *decoder*, would be constructed to model the desired input distribution. The decoder (NN) takes in as input a noise vector or a sampled latent variable **z** and maps it to the parameters of a probability distribution, such a mean and covariance of a multivariate Gaussian, or, as in this paper, the mean of a multivariate Bernoulli distribution, i.e., **z**^0^ = NN(**z**) where ${{{{{{{{\bf{x}}}}}}}}}_{i} \sim B(n=1,{{{{{{{\bf{p}}}}}}}}={{{{{{{{\bf{z}}}}}}}}}_{i}^{0})$. This artificial neural network would typically be made up of *L* + 1 layers of neurons, or *L* layers of hidden neurons and one output layer of neurons, where the state in layer *ℓ* is represented by a vector **z**^*ℓ*^. Each layer *ℓ* is interpreted as a transformation of the layer before it. In essence, **z**^*ℓ*−1^ = *ϕ*^*ℓ*−1^(**W**^*ℓ*^ ⋅ **z**^*ℓ*^) where **z**^*L*^ of layer *L* is set to be the same as the input noise vector **z**. The output **z**^0^ of this decoder (Fig. 1a) would be the parameters of a probability distribution, such as the mean of a Bernoulli distribution, or mean and covariance of a Gaussian (see Supplementary Note 7 for descriptions of the backprop-based networks used in this study). One can sample from this distribution to get a sample point **x** or use the mean of the distribution directly. To stabilize and speed up the model’s learning process, an encoder is typically introduced which also takes in as input the sensory input **x** to be predicted. The encoder is designed to drive the parameters of a distribution, normally a multivariate Gaussian, that shapes and controls the form of the latent variable **z**, i.e., ${\mu }_{z},{\sigma }_{z}^{2}={{{\mbox{NN}}}}_{e}({{{{{{{\bf{x}}}}}}}})$ where ${\sigma }_{z}^{2}={{{{{{{{\boldsymbol{\Sigma }}}}}}}}}_{z}\odot {{{{{{{\bf{I}}}}}}}}$ (or diagonal covariance).

To fit this model to the data, one would choose the weight parameters **W**^*ℓ*^ to minimize a loss function *ψ* such as the negative log-likelihood, typically using some variant of stochastic gradient descent. Often the backprop algorithm is used to compute the partial derivatives of $\frac{\partial \psi }{\partial {{{{{{{{\bf{W}}}}}}}}}^{\ell }}$ needed for this optimization. Computing the necessary derivatives according to backprop entails first computing an error signal at the output (downstream) layer, or ${{{{{{{{\bf{e}}}}}}}}}^{0}=\frac{\partial \psi }{\partial {{{{{{{{\bf{z}}}}}}}}}^{0}}$. This error signal is then transmitted to internal (upstream) neurons by carrying this signal back along the forward synapses that were originally used to transform **z**^*L*^—this is done by multiplying the signal with the transpose of the forward weight matrices. Furthermore, knowledge of the derivative of each activation function *ϕ*^*ℓ*^ is required during these computations (as shown in Fig. 1a).

### Backprop-learning versus brain-like learning

While the backprop algorithm described above has proven to be popular and effective in training ANNs, including generative models^33^, it has certain mechanisms that differ from the current understanding of brain-like learning. For example, in backprop:

1. Synapses that make up the forward information pathway need to directly be used in reverse to communicate teaching signals, i.e., the weight-transport problem^13^,
2. Neurons need to know about and communicate their own activation function’s first derivative,
3. Neurons must wait for the neurons ahead of them to percolate their own error signals backwards before they know when/how to adjust their own synapses, i.e., the update-locking problem^34^,
4. There is a distinct form of information propagation for error feedback, one that starts from the system’s output and works its way back to the input layer (see Fig. 1a), which does not influence neural activity, i.e., the global feedback pathway problem^12^ (backprop creates signals that only affect weights but do not, at least directly, affect/improve the network’s representations of the environment),
5. The error signals have a one-to-one correspondence with neurons.

The goal of this paper is to present a modeling and learning framework that uses fewer mechanisms that are incompatible with current understanding of brain-like learning. Specifically, we aim to address the first four items.

### The neural generative coding framework

**Fig. 2:**
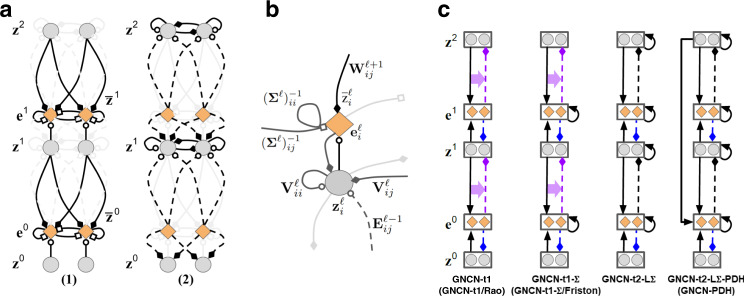
Neural generative coding computation and circuitry. **a** The two key computation steps taken by an entire NGC network (a GNCN-t2-LΣ) when processing an input (**z**^0^ = **x**): (1) prediction and laterally-weighted error computation, (2) error-correction of neural states. In this diagram, we depict a toy network with 3 layers of 2 state neurons (gray circles), i.e., ${{{{{{{{\bf{z}}}}}}}}}^{2}={([{z}_{0}^{2},{z}_{1}^{2}])}^{T}$, ${{{{{{{{\bf{z}}}}}}}}}^{1}={([{z}_{0}^{1},{z}_{1}^{1}])}^{T}$, ${{{{{{{{\bf{z}}}}}}}}}^{0}={([{z}_{0}^{0},{z}_{1}^{0}])}^{T}$, that are updated iteratively over *T* time steps. Two of these layers are linked to error neurons (orange diamonds), i.e., ${{{{{{{{\bf{e}}}}}}}}}^{1}={([{e}_{0}^{1},{e}_{1}^{1}])}^{T}$, ${{{{{{{{\bf{e}}}}}}}}}^{0}={([{e}_{0}^{0},{e}_{1}^{0}])}^{T}$, which compute mismatch messages that are propagated throughout the system. The bottom layer **z**^0^ receives sensory input, i.e., an image. **b** The basic neural computational unit that an NGC model is composed of, consisting of a single state neuron ${z}_{i}^{\ell }$ and an error neuron ${e}_{i}^{\ell }$ at layer *ℓ*. In the circuit, observe that a state neuron not only receives messages from error neurons (carried by **E**^*ℓ*−1^ synapses) but also a self-excitation signal and inhibition signals from laterally connected neurons (via **V**^*ℓ*^ synapses). The error neuron receives a gain signal (via ${({{{{{{{{\boldsymbol{\Sigma }}}}}}}}}^{\ell })}^{-1}$ synapses) from laterally connected error neurons. Note that filled diamonds indicate inhibitory signals, non-filled circles indicate excitatory signals, and empty squares indicate multiplicative signals. **c** The different GNCN architectures (under the NGC framework) experimented with in this study. Solid black arrow represents generative weights, dashed black arrow represents error weights, and dashed light pink arrow represents a temporary (virtual) backwards error pathway that is a function of the generative weights, i.e., the transpose of the error weights (the horizontal solid pink arrow indicates which forward weights are used to create the backward path weights).

In contrast to the backprop-based way of designing and training ANN generative models, our proposed framework, neural generative coding (NGC, see Fig. 1b), provides one way to emulate the several neurobiological principles and properties described above by proposing a family of models and their corresponding training procedure. In this framework, any single model is referred to as a generative neural coding network (GNCN, see Supplementary Note 1 for naming convention details). A GNCN model has *L* + 1 layers of neurons (also called state variables) ${{\mathfrak{N}}}^{0},{{\mathfrak{N}}}^{1},\ldots ,{{\mathfrak{N}}}^{L}$, where ${{\mathfrak{N}}}^{0}$ is the output layer. Each layer ${{\mathfrak{N}}}^{\ell }$ has *J*^*ℓ*^ neurons and each neuron has a latent state value represented by a single number. The combined latent state of all neurons in layer ${{\mathfrak{N}}}^{\ell }$ is represented by the vector ${{{{{{{{\bf{z}}}}}}}}}^{\ell }\in {{{{{{{{\mathcal{R}}}}}}}}}^{{J}_{\ell }\times 1}$ (initially **z**^*ℓ*^ = **0** when a new data point is encountered), with **z**^0^ being the same as the data **x** (it is typically clamped to the input, i.e., **z**^0^ = **x**). The network is interpreted as a specification of the probability *P*(**z**^0^ = **x**, **z**^1^, ⋯ , **z**^*L*^) = *P*(**z**^0^∣**z**^1^) ⋯ *P*(**z**^*L*−1^∣**z**^*L*^)*P*(**z**^*L*^), similar to a Bayesian network, and we use the notation **Z** = {**z**^1^, ⋯ , **z**^*L*^} to refer to the state of all of the *intermediate* neurons (i.e., excluding the output). Thus, layer ${{\mathfrak{N}}}^{\ell }$ represents the conditional probability *P*(**z**^*ℓ*^∣**z**^*ℓ*+1^), with the last layer ${{\mathfrak{N}}}^{L}$ representing *P*(**z**^*L*^). For image data, the distribution *P*(**z**^0^∣**z**^1^) associated with the output layer is multivariate Bernoulli with mean vector ${\overline{{{{{{{{\bf{z}}}}}}}}}}^{0}$ (which depends on **z**^1^). All of the other distributions *P*(**z**^*ℓ*^∣**z**^*ℓ*+1^) are multivariate Gaussians with mean vector ${\overline{{{{{{{{\bf{z}}}}}}}}}}^{\ell }$ (which depends on **z**^*ℓ*+1^) and covariance matrix **Σ**^*ℓ*^. The mean vector ${\overline{{{{{{{{\bf{z}}}}}}}}}}^{\ell }$ for layer ${{\mathfrak{N}}}^{\ell }$ is obtained in a feed-forward manner from the latent state of the neighboring layer (biases/offset terms have been omitted for clarity). Specifically, we model this generative process with the following equation:

1 $${\overline{{{{{{{{\bf{z}}}}}}}}}}^{\ell }\leftarrow {g}^{\ell }\left({\overbrace{{{{{{{{{\bf{W}}}}}}}}}^{\ell +1}\cdot {\phi }^{\ell +1}({{{{{{{{\bf{z}}}}}}}}}^{\ell +1})}^{\begin{array}{c}\scriptstyle{{{{{{{\rm{local}}}}}}}},\,{{{{{{{\rm{top}}}}}}}}-{{{{{{{\rm{down}}}}}}}}\,{{{{{{{\rm{prediction}}}}}}}}\end{array}}}+{\alpha }_{m}{\overbrace{\left({{{{{{{{\bf{M}}}}}}}}}^{\ell +2}\cdot {\phi }^{\ell +2}({{{{{{{{\bf{z}}}}}}}}}^{\ell +2})\right)}^{\begin{array}{c}\scriptstyle{{{{{{{\rm{local}}}}}}}},\,{{{{{{{\rm{auxiliary}}}}}}}}\,{{{{{{{\rm{prediction}}}}}}}}\end{array}}}\right),$$

where **W**^*ℓ*^ is a forward/generative weight matrix, *α*_*m*_ = 0, *g*^*ℓ*^, and *ϕ*^*ℓ*+1^ are activation functions, and ⋅ indicates matrix multiplication. Thus each layer ${{\mathfrak{N}}}^{\ell }$ is specified by two functions *g*^*ℓ*^ and *ϕ*^*ℓ*^, a trainable weight matrix **W**^*ℓ*^ and a covariance matrix **Σ**^*ℓ*^ (the last layer ${{\mathfrak{N}}}^{L}$ is specified just by the activation function *ϕ*^*L*^). Note in Eq. (1), the mean vector ${\overline{{{{{{{{\bf{z}}}}}}}}}}^{\ell }$ depends on the sampled realization of **z**^*ℓ*+1^ from the previous layer, making this a hierarchical, Gaussian latent variable model. Notice that, optionally, if the binary coefficent *α*_*m*_ is set to one, each layer also incorporates a learnable auxiliary generative matrix **M**^*ℓ*+2^, which conveys and injects state value information from the layer ${{\mathfrak{N}}}^{\ell +2}$ into the prediction of layer ${{\mathfrak{N}}}^{\ell }$ through a linear combination. We append the suffix “-PDH” (partially decomposable hierarchy) to a GNCN model name when *α*_*m*_ = 1 (see Fig. 2c for a visual depiction).

The goal of any GNCN model is to learn a joint distribution over its *L* + 1 neural states, i.e., *p*(**z**^0^, **z**^1^, ⋯, **z**^*L*^), from which the marginal distribution of the data may be obtained via *p*(**x**) = ∫_**Z**_*p*(**x**, **z**^1^, ⋯, **z**^*L*^)*d***Z** = ∫_**Z**_*p*(**z**^0^, **z**^1^, ⋯, **z**^*L*^)*d***Z**. Given that minimizing $-\log p({{{{{{{\bf{x}}}}}}}})$ directly is intractable in general, our approach for training is to approximately minimize the log-likelihood based on the ideas behind the Expectation-Maximization (EM) algorithm. Specifically, we work with the analog of the *complete-data* likelihood, which adds in the latent variables of the network (it does not marginalize over them) and sets up a 2 step process that adjusts the latent variables (like an E-step) and then updates the network parameters (M step).

### Training the model

The complete data log-likelihood *ψ* (also referred to as total discrepancy^2^) of the observed data **x** and the latent variables **z**^1^, …, **z**^*L*^ is defined formally as follows:

2 $$\psi =	\, \mathop{\sum}\limits_{j}\left({{{{{{{{\bf{x}}}}}}}}}_{j}\log {\overline{{{{{{{{\bf{z}}}}}}}}}}_{j}^{0}+(1-{{{{{{{{\bf{x}}}}}}}}}_{j})\log (1-{\overline{{{{{{{{\bf{z}}}}}}}}}}_{j}^{0})\right)\\ 	+\mathop{\sum }\limits_{\ell =1}^{L}\left(-\frac{1}{2}\log | {({{{{{{{{\boldsymbol{\Sigma }}}}}}}}}^{\ell })}^{-1}| -\frac{1}{2}{({{{{{{{{\bf{z}}}}}}}}}^{\ell }-{\overline{{{{{{{{\bf{z}}}}}}}}}}^{\ell })}^{T}\cdot {({{{{{{{{\boldsymbol{\Sigma }}}}}}}}}^{\ell })}^{-1}\cdot ({{{{{{{{\bf{z}}}}}}}}}^{\ell }-{\overline{{{{{{{{\bf{z}}}}}}}}}}^{\ell })\right).$$

Importantly, the above complete data log-likelihood connects our NGC models (and total discrepancy) to the principle of free energy^1,5^, given that the sum of (weighted) prediction errors defined in Eq. (2) can be shown to be a form of free energy that is minimized through variational inference. Explicitly characterizing a neural system engaged with optimizing the above objective as a generative model, as we do in this paper, grounds NGC as performing a form of approximate Bayesian inference (much as^5^ did for the early predictive coding model of ref. ^32^) and connects it to perception as (unconscious) inference^35^ and the larger theoretical framework of the Bayesian brain^36,37^.

Since all of the latent variables are continuous, the updates below follow the form of the exact gradient, i.e., differentiation (allowing for gradient descent), to optimize the latent variables and the parameters. The log-likelihood has the following partial derivatives:

3 $$\frac{\partial \psi }{\partial {({{{{{{{{\boldsymbol{\Sigma }}}}}}}}}^{\ell })}^{-1}}=\frac{1}{2}{{{{{{{{\boldsymbol{\Sigma }}}}}}}}}^{\ell }-\frac{1}{2}({{{{{{{{\bf{z}}}}}}}}}^{\ell }-{\overline{{{{{{{{\bf{z}}}}}}}}}}^{\ell })\cdot {({{{{{{{{\bf{z}}}}}}}}}^{\ell }-{\overline{{{{{{{{\bf{z}}}}}}}}}}^{\ell })}^{T}$$

4 $$\frac{\partial \psi }{\partial {{{{{{{{\boldsymbol{\Sigma }}}}}}}}}^{\ell }}=-\frac{1}{2}{{{{{{{{\boldsymbol{\Sigma }}}}}}}}}^{-1}+\frac{1}{2}{{{{{{{{\boldsymbol{\Sigma }}}}}}}}}^{-1}\cdot ({{{{{{{{\bf{z}}}}}}}}}^{\ell }-{\overline{{{{{{{{\bf{z}}}}}}}}}}^{\ell })\cdot {({{{{{{{{\bf{z}}}}}}}}}^{\ell }-{\overline{{{{{{{{\bf{z}}}}}}}}}}^{\ell })}^{T}\cdot {{{{{{{{\boldsymbol{\Sigma }}}}}}}}}^{-1}$$

5 $$\frac{\partial \psi }{\partial {{{{{{{{\bf{W}}}}}}}}}^{0}}=\left(\frac{\partial {g}^{0}}{\partial {{{{{{{{\bf{h}}}}}}}}}^{0}}\odot ({{{{{{{\bf{x}}}}}}}}\oslash {\overline{{{{{{{{\bf{z}}}}}}}}}}^{0}-({{{{{{{\bf{1}}}}}}}}-{{{{{{{\bf{x}}}}}}}})\oslash ({{{{{{{\bf{1}}}}}}}}-{\overline{{{{{{{{\bf{z}}}}}}}}}}^{0}))\right)\cdot {\left({\phi }^{1}({{{{{{{{\bf{z}}}}}}}}}^{1})\right)}^{T}$$

where ⊘ is element-wise division, ⊙ is element-wise product, and **h**^*ℓ*^ = **W**^*ℓ*^ ⋅ *ϕ*^*ℓ*+1^(**z**^*ℓ*+1^)

6 $$\frac{\partial \psi }{\partial {{{{{{{{\bf{W}}}}}}}}}^{\ell }}=\frac{\partial \psi }{\partial {{{{{{{{\bf{h}}}}}}}}}^{\ell }}\cdot {\left({\phi }^{\ell +1}({{{{{{{{\bf{z}}}}}}}}}^{\ell +1})\right)}^{T}$$

7 $$\frac{\partial \psi }{\partial {{{{{{{{\bf{z}}}}}}}}}^{1}}=\frac{\partial }{\partial {{{{{{{{\bf{z}}}}}}}}}^{1}}\left(\mathop{\sum}\limits_{j}\left({{{{{{{{\bf{x}}}}}}}}}_{j}\log {\overline{{{{{{{{\bf{z}}}}}}}}}}_{j}^{0}+(1-{{{{{{{{\bf{x}}}}}}}}}_{j})\log (1-{\overline{{{{{{{{\bf{z}}}}}}}}}}_{j}^{0})\right)\right)-{({{{{{{{{\boldsymbol{\Sigma }}}}}}}}}^{\ell })}^{-1}\cdot ({{{{{{{{\bf{z}}}}}}}}}^{1}-{\overline{{{{{{{{\bf{z}}}}}}}}}}^{1})$$

8 $$\frac{\partial \psi }{\partial {{{{{{{{\bf{z}}}}}}}}}^{\ell }}=\left(\frac{\partial {\overline{{{{{{{{\bf{z}}}}}}}}}}^{\ell -1}}{\partial {{{{{{{{\bf{z}}}}}}}}}^{\ell }}\cdot \left({({{{{{{{{\boldsymbol{\Sigma }}}}}}}}}^{\ell -1})}^{-1}\cdot ({{{{{{{{\bf{z}}}}}}}}}^{\ell -1}-{\overline{{{{{{{{\bf{z}}}}}}}}}}^{\ell -1})\right)\right)-{({{{{{{{{\boldsymbol{\Sigma }}}}}}}}}^{\ell })}^{-1}\cdot ({{{{{{{{\bf{z}}}}}}}}}^{\ell }-{\overline{{{{{{{{\bf{z}}}}}}}}}}^{\ell }).$$

In this work, we incorporate two key concepts from local representation alignment (LRA)^12,38^: (1) the use of error synapses to directly resolve the weight-transport problem, and (2) the omission of derivatives of activation functions which yield synapse rules that function like error-Hebbian updates. To incorporate these modifications, we introduce a vector of error neurons **e**^*ℓ*^ (depicted in Fig. 1b) that are tasked with computing how far off the mean vector is from the relevant nearby state. Note that the error neurons themselves are derived from the likelihood function:

9 $${{{{{{{{\bf{e}}}}}}}}}^{0}=\frac{\partial \psi }{\partial {\overline{{{{{{{{\bf{z}}}}}}}}}}^{0}}=({{{{{{{\bf{x}}}}}}}}\oslash {\overline{{{{{{{{\bf{z}}}}}}}}}}^{0}-({{{{{{{\bf{1}}}}}}}}-{{{{{{{\bf{x}}}}}}}})\oslash ({{{{{{{\bf{1}}}}}}}}-{\overline{{{{{{{{\bf{z}}}}}}}}}}^{0}))$$

10 $${{{{{{{{\bf{e}}}}}}}}}^{\ell }=\frac{\partial \psi }{\partial {\overline{{{{{{{{\bf{z}}}}}}}}}}^{\ell }}={\overbrace{{({{{{{{{{\boldsymbol{\Sigma }}}}}}}}}^{\ell -1})}^{-1}}^{\begin{array}{c}\scriptstyle{{{{{{{\rm{lateral}}}}}}}}\,{{{{{{{\rm{modulation}}}}}}}}\end{array}}}\cdot\, {\overbrace{({{{{{{{{\bf{z}}}}}}}}}^{\ell -1}-{\overline{{{{{{{{\bf{z}}}}}}}}}}^{\ell -1})}^{\begin{array}{c}\scriptstyle{{{{{{{\rm{mismatch}}}}}}}}\,{{{{{{{\rm{signal}}}}}}}}\end{array}}}$$

and are implemented as separate sets of activities (like the state neurons)—note that Eq. (9) simplifies further if ${g}^{0}({z}_{i}^{0})=1/\left(1+\exp (-{z}_{i}^{0})\right.$ to ${{{{{{{{\bf{e}}}}}}}}}^{0}={{{{{{{\bf{x}}}}}}}}-{\overline{{{{{{{{\bf{z}}}}}}}}}}^{0}$. In Fig. 2b, we depict the circuit for a single pair of neurons at a layer ${{\mathfrak{N}}}^{\ell }$, i.e., a state neuron ${{{{{{{{\bf{z}}}}}}}}}_{i}^{\ell }$ and an error neuron ${{{{{{{{\bf{e}}}}}}}}}_{i}^{\ell }$. Notice that, in Eq. (10) above, in order to compute the state of the error neuron ${e}_{i}^{\ell }$, the covariance matrix **Σ**^*ℓ*^ acts as a lateral modulation matrix, which is inspired by the neuro-mechanistic concept of precision weighting in predictive processing theory^39^. Specifically, it allows the error neuron ${e}_{i}^{\ell }$ to dynamically amplify/reduce the learning signal (i.e., ${z}_{i}^{\ell }-{\bar{z}}_{i}^{\ell }$) of its corresponding state neuron ${z}_{i}^{\ell }$, based on the learning signals of the other state neurons in the same layer. Empirically, we found these modulatory synapses to improve the crispness of the final model samples. Note that Eq. (10) is applied to the *J*_*ℓ*_ error neurons in each layer *ℓ* = 1, ⋯, *L* − 1.

### Updating the states and synapses

To transmit the error neuron activity values needed to calculate the update to state **z**^*ℓ*^, we replace the term $\frac{\partial {{{{{{{{\bf{z}}}}}}}}}^{\ell -1}}{\partial {{{{{{{{\bf{z}}}}}}}}}^{\ell }}$ with a learnable error matrix **E**^*ℓ*^. This substitution allows us to rewrite Eqs. (7) and (8) as follows:

11 $$\frac{\partial \psi }{\partial {{{{{{{{\bf{z}}}}}}}}}^{1}}\approx {{\Delta }}{{{{{{{{\bf{z}}}}}}}}}^{1}={{{{{{{{\bf{E}}}}}}}}}^{1}\cdot \left(\frac{{g}^{0}}{{{{{{{{{\bf{h}}}}}}}}}^{0}}\odot {{{{{{{{\bf{e}}}}}}}}}^{0}\right)-{{{{{{{{\bf{e}}}}}}}}}^{1}={{{{{{{{\bf{E}}}}}}}}}^{1}\cdot \left({{{{{{{{\bf{z}}}}}}}}}^{0}-{\overline{{{{{{{{\bf{z}}}}}}}}}}^{0}\right)-{{{{{{{{\bf{e}}}}}}}}}^{1}$$

12 $$\frac{\partial \psi }{\partial {{{{{{{{\bf{z}}}}}}}}}^{\ell }}\approx {{\Delta }}{{{{{{{{\bf{z}}}}}}}}}^{\ell }={{{{{{{{\bf{E}}}}}}}}}^{\ell }\cdot {{{{{{{{\bf{e}}}}}}}}}^{\ell -1}-{{{{{{{{\bf{e}}}}}}}}}^{\ell }.$$

Once the update for any layer ${{\mathfrak{N}}}^{\ell }$ has been calculated, the corresponding state neurons will proceed to update their state values. By analogy with latent variable models, where the state neurons **z**^*ℓ*^ correspond to latent variables, this act can be viewed as an attempt to modify the states in a way that improves the complete data log-likelihood (i.e., modifying the **z**^*ℓ*^ to cause *p*(**x**, **z**^1^, ⋯, **z**^*L*^) to increase). One possible neuroscience-inspired way to perform this update is shown in Eq. (13) (here *β* is a hyperparameter akin to the machine learning concept of a learning rate). Specifically, this update is:

13 $${{{{{{{{\bf{z}}}}}}}}}_{i}^{\ell }\leftarrow {{{{{{{{\bf{z}}}}}}}}}_{i}^{\ell }+\beta \left(-\gamma {{{{{{{{\bf{z}}}}}}}}}_{i}^{\ell }+{\overbrace{{\sum }_{j\in {J}_{\ell -1}}({{{{{{{{\bf{E}}}}}}}}}_{ij}^{\ell }{{{{{{{{\bf{e}}}}}}}}}_{j}^{\ell -1})-{{{{{{{{\bf{e}}}}}}}}}_{i}^{\ell }}^{\begin{array}{c}{{\Delta }}{{{{{{{{\bf{z}}}}}}}}}_{i}\end{array}}}\right).$$

Here ${z}_{i}^{\ell }$ is modified through three terms. The first is a decaying pressure caused by the leak term $-{{{\bf{z}}}}_{i}^{\ell }$, controlled by the strength factor *γ*. The second term, $-{{{{{{{{\bf{e}}}}}}}}}_{i}^{\ell }$, can be interpreted as top-down pressure where ${{{{{{{{\bf{e}}}}}}}}}_{i}^{\ell }$ is a measure of how much the *i*th neuron’s state differs from the predicted state value ${\overline{{{{{{{{\bf{z}}}}}}}}}}_{i}^{\ell }$ that is computed by the layer above. Finally, the third term adds in the error message from each error neuron ${{{{{{{{\bf{e}}}}}}}}}_{j}^{\ell -1}$ in the layer below, communicated by special error synapses **E**^*ℓ*^, acting as a form of bottom-up pressure. It is important to mention that, in this paper, we investigated another particular form of Eq. (13) as follows:

14 $${{{{{{{{\bf{z}}}}}}}}}_{i}^{\ell }\leftarrow {{{{{{{{\bf{z}}}}}}}}}_{i}^{\ell }+\beta \left(-\gamma {{{{{{{{\bf{z}}}}}}}}}_{i}^{\ell }+\frac{\partial \phi ({{{{{{{{\bf{z}}}}}}}}}_{i}^{\ell })}{\partial {{{{{{{{\bf{z}}}}}}}}}_{i}^{\ell }}\left[\mathop{\sum}\limits_{\,j\in {J}_{\ell -1}}\left({{{{{{{{\bf{U}}}}}}}}}_{ij}^{\ell }{{{{{{{{\bf{e}}}}}}}}}_{j}^{\ell -1}\right)-{{{{{{{{\bf{e}}}}}}}}}_{i}^{\ell }\right]-\lambda {{{{{{{\rm{sign}}}}}}}}({{{{{{{{\bf{z}}}}}}}}}_{i})\right).$$

where the last term −*λ*sign(**z**_*i*_) is a kurtotic prior that can be imposed to encourage most activity values to be closer to zero within a given neural state **z**^*ℓ*^. Under the NGC framework, models that use separate, learnable error synapses **E**^*ℓ*^ will be referred to as “Type 2” (t2) and those that use (non-learnable, virtual) error synapses that are a function of the forward weights **W**^*ℓ*^ will be labeled as “Type 1” (t1) (see Fig. 2c for visual depictions of these models and Supplementary Note 1 for details on the NGC framework, the naming convention, and other alternative possible structures). We can then manipulate certain variable values to recover different classical predictive coding models: (1) if *γ* = 0, **U** = (**W**)^*T*^, ${{{{{{{{\boldsymbol{\Sigma }}}}}}}}}^{\ell }={\sigma }_{z}^{2}{{{{{{{\bf{I}}}}}}}}$, and ${\phi }^{\ell }(v)=\tanh (v)$, then we recover the model proposed in ref. ^32^—we will refer to this as GNCN-t1/Rao (using *γ* > 0 yields the state equation of ref. ^40^), and (2) if *γ* = 0, **U** = −(**W**)^*T*^, and ${\phi }^{\ell }(v)=\max (0,v)$, then we recover the neural implementation of the graphical model proposed in ref. ^5^—we will refer to this as GNCN-t1-Σ/Friston (see Fig. 2c).

However, instead of directly using the update rule in Eq. (13), we may further incorporate another property of the brain—activation sparsity (through lateral inhibition/excitation). Sparsity in real neuronal and artificial systems is often argued to be useful for learning compact representations (most activity values will be at or near zero), allowing for efficient storage and vastly improved energy efficiency. To emulate this type of sparsity, we integrate an explicit mechanism to force neurons to compete for activation (in contrast to the kurtotic prior used in Eq. (14)), where we take inspiration from the known occurrence of lateral synapses in cortical regions of the brain (which are thought to facilitate contextual processing^41^). To do this, we introduce two terms to Eq. (13) that use excitatory/inhibitory synapses stored in a matrix **V**^*ℓ*^. This means that state neurons are updated as follows:

15 $${{{{{{{{\bf{z}}}}}}}}}_{i}^{\ell }\leftarrow {{{{{{{{\bf{z}}}}}}}}}_{i}^{\ell }+\beta \left({\overbrace{-\gamma {{{{{{{{\bf{z}}}}}}}}}_{i}^{\ell }}^{\begin{array}{c}\scriptstyle{{{{{{{\rm{leak}}}}}}}}\end{array}}}+{\overbrace{\left(\mathop{\sum}\limits_{j\in {J}_{\ell -1}}{{{{{{{{\bf{E}}}}}}}}}_{ij}^{\ell }{{{{{{{{\bf{e}}}}}}}}}_{j}^{\ell -1}\right)-{{{{{{{{\bf{e}}}}}}}}}_{i}^{\ell }}^{\begin{array}{c}\scriptstyle{{{{{{{\rm{bottom}}}}}}}}{\mbox{-}}{{{{{{{\rm{up}}}}}}}}+{{{{{{{\rm{top}}}}}}}}{\mbox{-}}{{{{{{{\rm{down}}}}}}}}\,{{{{{{{\rm{pressures}}}}}}}}\end{array}}}-{\overbrace{\left(\mathop{\sum}\limits_{j\in {J}_{\ell },j\ne i}{{{{{{{{\bf{V}}}}}}}}}_{ij}^{\ell }{\phi }^{\ell }({{{{{{{{\bf{z}}}}}}}}}_{j}^{\ell })\right)}^{\begin{array}{c}\scriptstyle{{{{{{{\rm{lateral}}}}}}}}\,{{{{{{{\rm{inhibition}}}}}}}}\end{array}}}+{\overbrace{{{{{{{{{\bf{V}}}}}}}}}_{ii}^{\ell }{\phi }^{\ell }({{{{{{{{\bf{z}}}}}}}}}_{i}^{\ell })}^{\begin{array}{c}\scriptstyle{{{{{{{\rm{self}}}}}}}}{\mbox{-}}{{{{{{{\rm{excitation}}}}}}}}\end{array}}}\right)$$

**Fig. 3:**
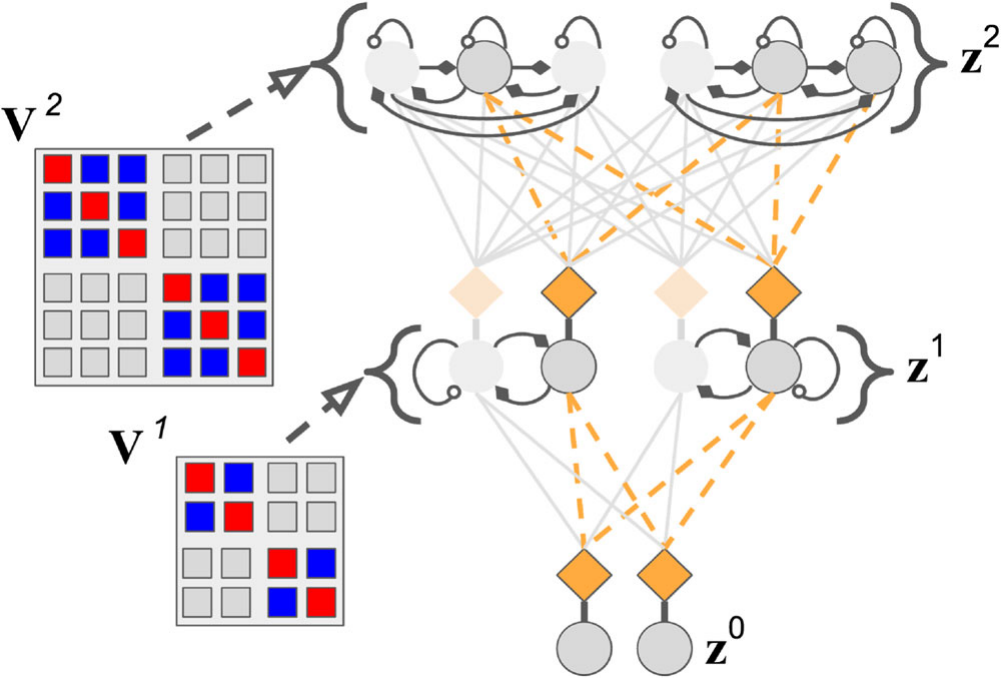
Lateral dynamics of generative neural coding. Depending on how the lateral connectivity matrices, **V**^1^ and **V**^2^, are designed, different competition patterns emerge among neurons in the network. In the system with lateral matrices (shown below), each neuron is driven by its own self-excitation (red-colored blocks) and laterally inhibits (blue-colored blocks) other neurons that inhabit the same group (of 2 or 3 units). In the figure, one possible outcome (or agent state) of such a competition is shown (at the end of a *T*-step stimulus presentation window). Self-excitation and lateral inhibition strengths are controlled by coefficients set a priori. Dashed orange edges show actively used synapses, filled diamonds indicate inhibitory signals, and non-filled circles indicate excitatory signals.

Depending on the values set in **V**^*ℓ*^, different types of sparsity patterns emerge, creating a flexible means for testing the benefits/drawbacks of different kinds of lateral competition patterns in an interpretable manner. Figure 3 provides a graphical example of the type of interaction pattern we found that worked well for the GNCN in this study, i.e., we forced *J*_*ℓ*_/*K* groups (or columns) of *K* neurons to compete with each other (see Supplementary Note 6). The model employing Eq. (15) with *α*_*m*_ = 0 (in its Eq. (1)) will be referred to as GNCN-t2-LΣ and when *α*_*m*_ = 1 (in its Eq. (1)) is used, the model will be referred to as the GNCN-PDH.

The synaptic matrix updates in Eqs. (5) and (6) can be written in terms of the error neurons:

16 $$\frac{\partial \psi }{\partial {{{{{{{{\bf{W}}}}}}}}}^{0}}\propto {{\Delta }}{{{{{{{{\bf{W}}}}}}}}}^{0}={{{{{{{{\bf{e}}}}}}}}}^{0}\cdot {({\phi }^{1}({{{{{{{{\bf{z}}}}}}}}}^{1}))}^{T}$$

17 $$\frac{\partial \psi }{\partial {{{{{{{{\bf{W}}}}}}}}}^{\ell }}\propto {{\Delta }}{{{{{{{{\bf{W}}}}}}}}}^{\ell }={{{{{{{{\bf{e}}}}}}}}}^{\ell }\cdot {({\phi }^{\ell +1}({{{{{{{{\bf{z}}}}}}}}}^{\ell +1}))}^{T}$$

and, following in line with LRA, the error synapses can be updated as:

18 $${{\Delta }}{{{{{{{{\bf{E}}}}}}}}}^{\ell }=\lambda \left({\phi }^{\ell +1}({{{{{{{{\bf{z}}}}}}}}}^{\ell +1})\cdot {({{{{{{{{\bf{e}}}}}}}}}^{\ell })}^{T}\right)$$

where *λ* ∈ [0, 1] controls the strength of the error synaptic adjustment (usually set to a value < 1). Note that no partial derivative of the activation function *ϕ*^*ℓ*^ is required (in Type 2 GNCN models)—we are using an error-driven, Hebbian-like update rule, which has been shown to be effective empirically and crucially removes the need for state neurons to be aware of their own point-wise activation derivative. Note that this means *ϕ*^*ℓ*^ could easily be a discrete function in this case, such as the signum or Heaviside.

Putting all of the components above together, given a data point(s) **x**, we can characterize the neural dynamics of the NGC system (graphically depicted in Fig. 2a) and train any GNCN following an online alternating maximization approach as follows:

// **Initialization:**

1. Set **z**^0^ = **x** (clamp data to output neurons), and set **z**^*ℓ*^ = **0**, ∀ *ℓ* ≥ 1
2. Compute mean estimates ${\overline{{{{{{{{\bf{z}}}}}}}}}}^{\ell },\ \forall \ell \ge 0$ (Eq. (1))
3. Use Eq. (9) and Eq. (10) to update (or initialize) all error neurons **e**^*ℓ*^, ∀ *ℓ* ≥ 0
  // **Latent Update Step:** Search for the most probable value of **z**^*ℓ*^, ∀  *ℓ*
4. Use Eq. (11) to get Δ**z**^1^ and Eq. (12) to get Δ**z**^*ℓ*^, ∀ *ℓ* > 1. States are modified via the vectorized form of Eq. (15):
  $${{{{{{{{\bf{z}}}}}}}}}^{1} ={{{{{{{{\bf{z}}}}}}}}}^{1}+\beta \left({{\Delta }}{{{{{{{{\bf{z}}}}}}}}}^{1}-{{{{{{{{\bf{V}}}}}}}}}^{1}\cdot {{{{{{{{\bf{z}}}}}}}}}^{1}-\gamma {{{{{{{{\bf{z}}}}}}}}}^{1}\right)\\ {{{{{{{{\bf{z}}}}}}}}}^{\ell } ={{{{{{{{\bf{z}}}}}}}}}^{\ell }+\beta \left({{\Delta }}{{{{{{{{\bf{z}}}}}}}}}^{\ell }-{{{{{{{{\bf{V}}}}}}}}}^{\ell }\cdot {{{{{{{{\bf{z}}}}}}}}}^{\ell }-\gamma {{{{{{{{\bf{z}}}}}}}}}^{\ell }\right),\ \forall \ell > 1$$
5. Repeat Steps 2, 3, and 4 for *T* iterations
  // **Parameter Update Step:** Update parameters given estimated latent states
6. Update synaptic matrices using Eqs. (16), (17), (3), and (18) (and normalize the matrices).

At test time, to reconstruct **x**, one should only use Steps 1–5 of the recipe above. *γ* controls the strength of the decay/leak applied to **z**^*ℓ*^ and corresponds to placing an additional ${{{{{{{\mathcal{N}}}}}}}}(\mu =0,{{\Sigma }}=\lambda {{{{{{{\bf{I}}}}}}}})$ prior over the latent states. In essence, the above complete process depicts that the GNCN adjusts it synaptic weight matrices once activity values for all **z**^*ℓ*^ and **e**^*ℓ*^ have been found after a *T*-step episode. The synaptic matrix updates are simply the products between relevant state activities and error neurons. Finally, after each weight update has been made, a GNCN’s weight matrices are normalized such that the Euclidean norms of their columns are 1.0 (this step, which requires non-local information to perform, could possibly be induced biologically through the use of external neuromodulatory signals). In essence, the steps described above and shown in Fig. 2a illustrate that the NGC framework embodies the idea that neural state and synaptic weight adjustment are the result of a process of generate-then-correct, or *continual error correction*, in response to collected samples of the agent’s environment.

### Generative neural coding learns viable auto-associative generative models

The model framework that we have described so far would be immediately useful for creating an auto-associative memory of sensory input, i.e., upon receiving a particular sensory input, the model would be able to recall seeing it by accurately reconstructing it. Furthermore, since the model is learning an estimator of the input distribution *p*(**x**), we may sample from it (see “Methods”) to synthesize or hallucinate data patterns, as we will demonstrate later in this section.

To evaluate our framework (see “Methods”), nine approaches were compared across four image datasets (Table 1), each of which contained a training subset (from which we created an additional validation subset) and a test subset (see “Methods”). One is a Gaussian mixture model (GMM) and eight are neural models—four of these are backprop-based (see Methods and Supplementary Note 7) and four are NGC models, i.e., a GNCN-t1/Rao (which is equivalent to the model of ref. ^32^ and ref. ^40^), a GNCN-t1-Σ/Friston (which is equivalent to the model of ref. ^5^), a GNCN-t2-LΣ, and a GNCN-PDH. All neural models were constrained to have their topmost layer to contain 20 processing elements (the GNCN-t2-LΣ and GNCN-PDH were restricted to 20 neural columns) and all had approximately the same total number of synapses. With respect to NGC models, *T* = 50 (see Supplementary Note 4). The regularized auto-encoder (RAE), the auto-associative network least equipped to serve as a data synthesizer, reaches lower reconstruction error, in terms of binary cross entropy (BCE), compared to the other backprop-based generative models, i.e., the Gaussian variational autoencoder (GVAE), the constant-variance GVAE (GVAE-CV), and the adversarial autoencoder (GAN-AE). However, while the GVAE-CV, GVAE, and GAN-AE models yield worse reconstruction than the RAE, they obtain much better log-likelihoods, especially compared to the GMM baseline, indicating that they are strong data samplers. It makes sense that these models obtain better likelihood at the expense of BCE given that their optimization objective imposes a strong pressure to craft a proper (Gaussian) distribution over latent variables in addition to reconstructing data samples (in the case of the GAN-AE, the pressure comes from forcing the discriminator to distinguish between fake and real latent variables). Interestingly enough, we see that the GNCN-t2-LΣ and GNCN-PDH obtain competitive log-likelihood with the GVAE-CV, GVAE, and GAN-AE with the GNCN-PDH yielding the best log-likelihood out of all GNCN models for all four datasets. Notably, the GNCN models result in the best reconstruction across all four datasets, with the GNCN-t1-Σ/Friston and GNCN-PDH offering the lowest BCE. On MNIST, we note that the GNCN-PDH outperforms some other related prior models^42^ that used our same experimental setup, e.g., a restricted Boltzmann machine with $\log p({{{{{{{\bf{x}}}}}}}})=-112$ nats, a denoising autoencoder with $\log p({{{{{{{\bf{x}}}}}}}})=-142$ nats (trained via backprop) and $\log p({{{{{{{\bf{x}}}}}}}})=-116$ nats (using the walk-back algorithm). As indicated by our results, NGC’s sampling and reconstruction ability are promising. Worthy of note, however, is that we find that backprop-based autoencoders appear to do better at matching the data’s class frequency than the GNCNs studied in this article (see Supplementary Note 3).

**Table 1:** Reconstruction and likelihood measurements: Generative modeling results across datasets.

Model	BCE	$\log p(x)$	BCE	$\log p(x)$
	MNIST		KMNIST	
GMM	–	− 185.37 ± 0.47	–	−426.73 ± 1.03
RAE	60.94 ± 0.58	− 117.01 ± 0.18	175.60 ± 0.51	−234.41 ± 1.17
GVAE-CV	69.90 ± 0.30	− 106.41 ± 0.06	180.81 ± 1.05	−229.12 ± 0.95
GVAE	75.52 ± 2.19	− 100.10 ± 2.26	199.37 ± 0.91	**−218.67** ± **0.43**
GAN-AE	73.54 ± 3.49	**−94.42** ± **0.07**	199.68 ± 3.01	**−216.76** ± **1.16**
GNCN-t1/Rao	66.47 ± 0.07	− 102.22 ± 0.02	136.49 ± 0.15	−226.37 ± 0.19
GNCN-t1-Σ	**47.25** **±** **0.19**	− 101.92 ± 0.24	**114.63** **±** **0.35**	−229.04 ± 0.52
GNCN-t2-LΣ	59.66 ± 0.02	− 98.27 ± 0.25	135.86 ± 0.17	−220.41 ± 0.22
GNCN-PDH	**44.86** **±** **0.05**	**−97.26** ± **0.08**	**128.39** **±** **0.23**	−219.82 ± 0.64
	FMNIST		CalTech	
GMM	–	−302.96 ± 0.47	–	−88.63 ± 0.43
RAE	102.72 ± 0.78	−138.76 ± 1.43	40.41 ± 0.76	−50.47 ± 2.07
GVAE-CV	111.71 ± 0.13	−131.82 ± 0.30	49.58 ± 4.59	−41.34 ± 0.84
GVAE	121.62 ± 0.45	**−127.64** ± **0.04**	38.35 ± 0.74	−44.07 ± 0.24
GAN-AE	132.94 ± 4.37	−130.87 ± 1.80	40.40 ± 0.74	**−40.68** **±** **0.13**
GNCN-t1/Rao	95.80 ± 0.03	−136.63 ± 0.47	34.60 ± 0.06	−44.62 ± 0.13
GNCN-t1-Σ	**81.73** **±** **0.42**	−133.51 ± 0.27	**31.17** **±** **0.02**	−42.53 ± 0.06
GNCN-t2-LΣ	97.80 ± 0.71	−132.04 ± 0.31	33.29 ± 0.01	−41.52 ± 0.06
GNCN-PDH	**90.06** **±** **0.38**	**−130.03** **±** **0.16**	**26.76** **±** **0.02**	**−40.82** **±** **0.20**

The binary cross entropy (BCE) of the model reconstructions and the marginal log-likelihood $\log p({{{{{{{\bf{x}}}}}}}})$ (in nats) on the test set are reported (lower BCE is better and $\log p({{{{{{{\bf{x}}}}}}}})$ closer to 0.0 is better). We set in bold font the best two scores across the set of models with respect to BCE (first column) as well as with respect to $\log p({{{{{{{\bf{x}}}}}}}})$ (second column). Metrics are averaged over 10 trials—we report their mean and standard deviation (note that GNCN-t1-Σ is the same as GNCN-t1-Σ/Friston and GNCN-t1 is the same GNCN-t1/Rao.).

**Fig. 4:**
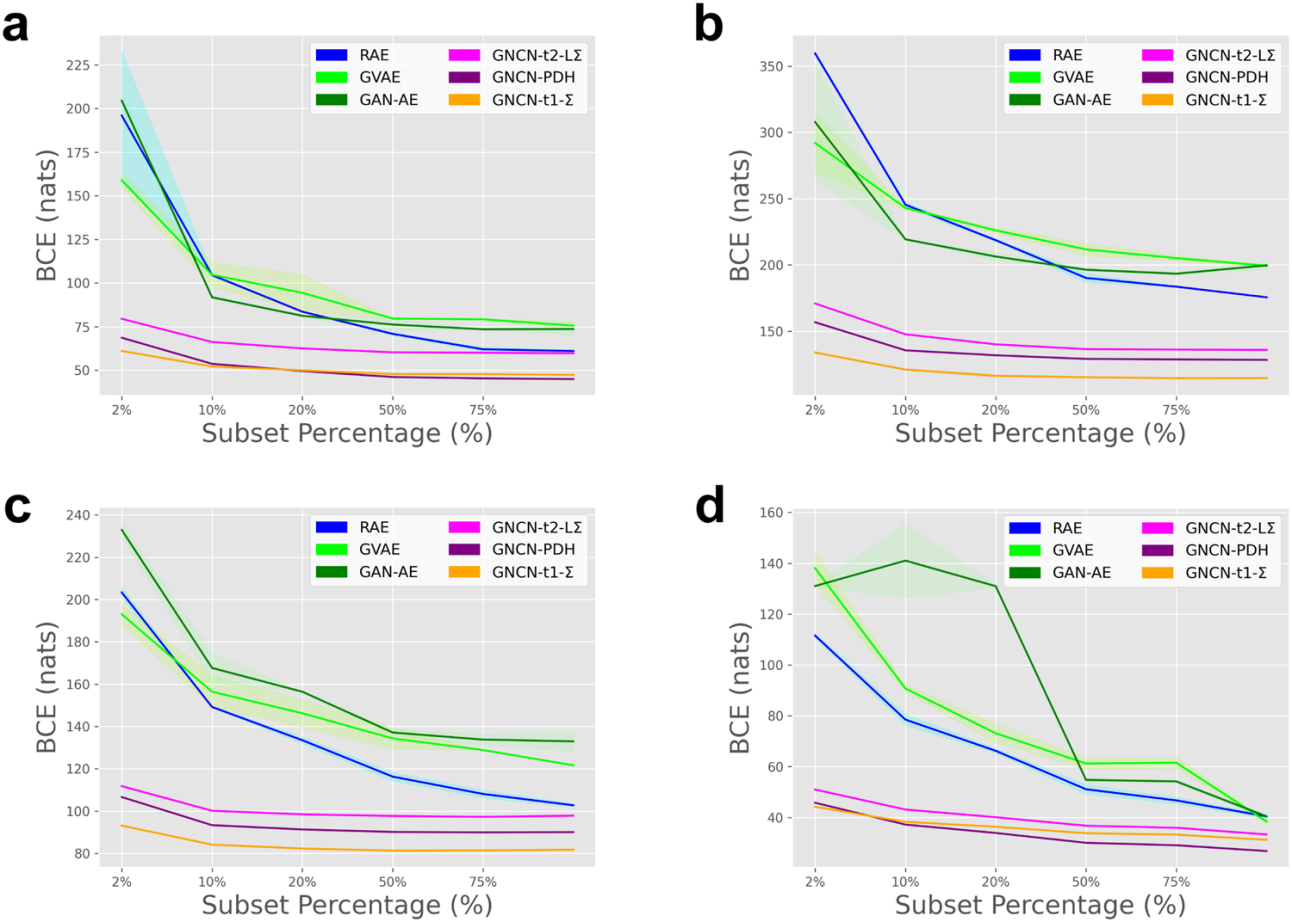
Data efficiency measurements. Test BCE loss measurements (lower is better) for pattern reconstruction on data subsets (measured in terms of percentage % of the original database sampled, with data points being extracted randomly without replacement) of increasing size for **a** MNIST, **b** KMNIST, **c** FMNIST, and **d** CalTech. Curves depict the mean (solid line) and the standard deviation (lighter-colored envelope) over 10 trials. Plotted are the resulting curves for three backprop-based autoencoder models, i.e., RAE, GVAE, and GAN-AE, and three neural generative coding models, i.e., GNCN-t2-LΣ, GNCN-PDH, and GNCN-t1-Σ. (Note that GNCN-t1-Σ is also referred to as GNCN-t1-Σ/Friston.).

**Fig. 5:**
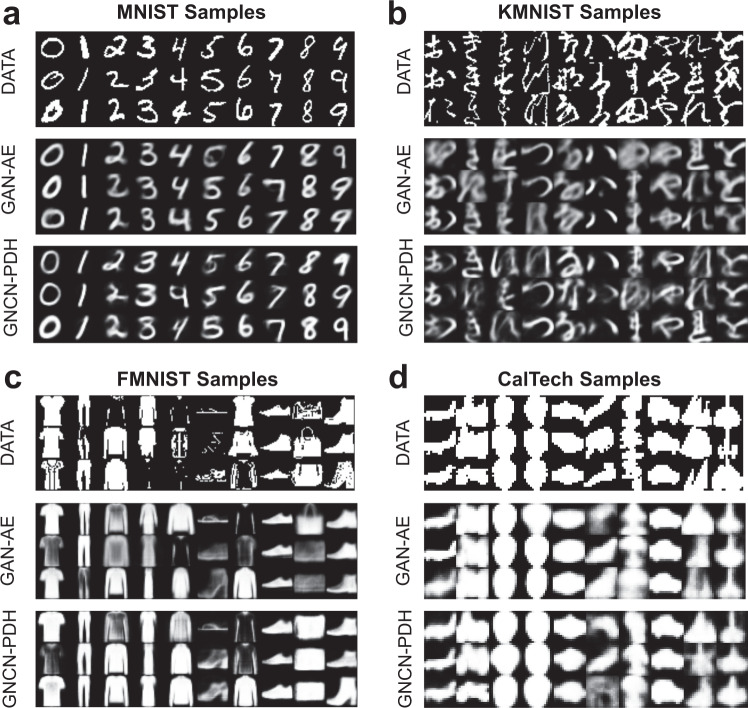
Model sample visualization. Shown are samples from models with overall best BCE-$\log p({{{{{{{\bf{x}}}}}}}})$ balance (each column corresponds to one class). Top row shows dataset samples (DATA), middle row shows adversarial autoencoder (GAN-AE), and bottom row shows neural generative coding (GNCN-PDH) samples. Columns are arranged, left to right, as: **a** MNIST, **b** KMNIST, **c** FMNIST, and **d** CalTech.

As shown in Fig. 4, we measured the data efficiency of the GNCN-t1-Σ/Friston, GNCN-t2-LΣ, and GNCN-PDH as well as several representative backprop-models (RAE, GVAE, and GAN-AE). Specifically, we train each model (to convergence) using a subset of the training data of varying size (we created six versions of each dataset’s training subset using either 2%, 10%, 20%, 50%, 75%, or 100% of the original sample), and plot final test BCE at convergence (versus percentage of original data used on the x-axis), for each of the models. Desirably, the result demonstrates that the NGC models generalize better given varying amounts of data, even with less data compared to the autoencoders. This benefit is useful given that it offsets the higher per-sample processing cost of the predictive processing models. Furthermore, in Fig. 5, we present random samples from each class obtained from either: (1) the original dataset, (2) ancestrally sampling the GAN-AE, or (3) ancestrally sampling the GNCN-PDH. Note that we trained a well-regularized MLP classifier on the original dataset and then used it to automatically annotate the samples produced by each model. Observe that both the GNCN-PDH and GAN-AE yield reasonably good-looking sample images for all four datasets and the differences in perceptual quality between the models’ sets of samples is marginal. This is encouraging, given that our goal was to demonstrate that an NGC model could be competitive with backprop-based generative models (see Supplementary Table 1 for additional visualization of nearest-neighbor samples that match an original data point for each class).

### Neural generative coding yields strong downstream pattern classifiers

All of the generative models that we have experimented with in this paper are unsupervised in nature, meaning that by attempting to learn a density estimator of the data’s underlying distribution, the representations acquired by each might prove useful for downstream applications, such as image categorization. To evaluate each how useful each model’s latent representations might be when attempting to discriminate between samples, we evaluate the performance of a simple log-linear classifier, i.e., maximum entropy, that is fit to each model’s topmost latent variable using the labels accompanying each dataset. For all models, we measure the classification error (Err), as a percentage, on the test set of each benchmark in Table 2, where the closer a model is to 0%, the better. In addition, we provide, for context, the results of a simple, purely discriminative baseline (the DSRN), which is simply a backprop-trained deep sparse recitfier network^43^ that is constrained to have the same number of synapses as the generative models. As we see in Table 2, in terms of test error, the NGC models (GNCN-t1/Rao, GNCN-t1-Σ/Friston, GNCN-t2-LΣ, and GNCN-PDH) are competitive with the purely discriminatively-trained DSRN and outperform all of the other generative models (even outperforming the DSRN in one out of the four cases).

**Table 2:**
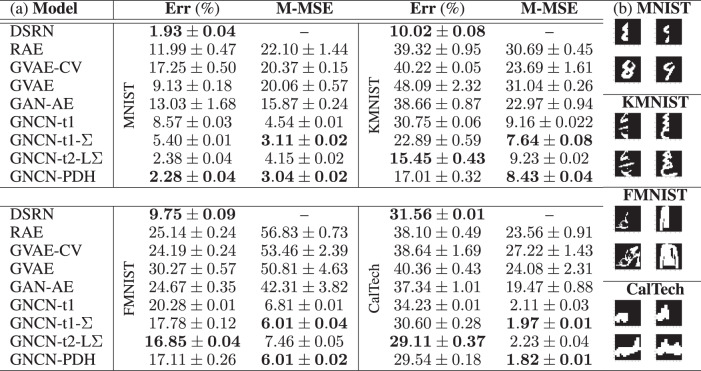
Downstream performance across datasets.

(a) The classification error (Err) of a log-linear model fit to each model’s latent codes and the masked mean squared error (M-MSE) of the model’s pattern completion ability on the test set are reported (lower is better—the two best scores are shown in bold font with respect to each metric/column). Metrics are averaged over 10 trials—we report their mean and standard deviation. (b) For each database, we present for MNIST, KMNIST, FMNIST, and CalTech (in order from top to bottom) two randomly selected image patterns (from each dataset’s training split), which are shown in the first row of each dataset’s table, and the GNCN-PDH’s completion of these patterns, displayed in the second row of each dataset’s table (note that GNCN-t1-Σ is the same as GNCN-t1-Σ/Friston and GNCN-t1 is the same GNCN-t1/Rao.).

**Fig. 6:**
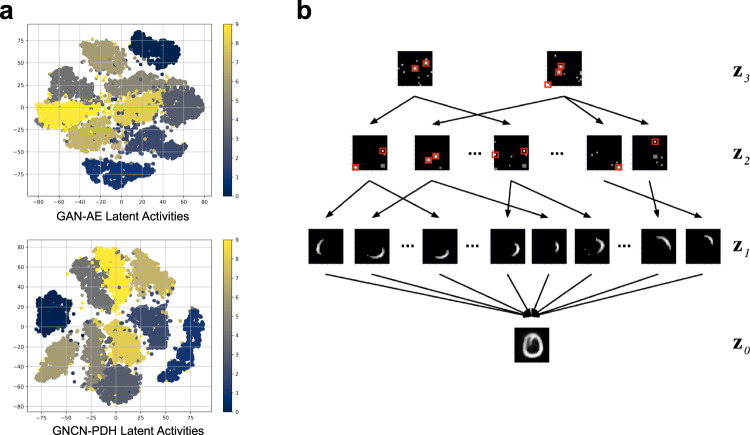
Examination of model latent activities. **a** t-SNE plot of latent codes of the GAN-AE and GNCN-PDH on the MNIST database. **b** Illustration of an NGC network composing the digit zero that was randomly sampled from the MNIST database (all the feature maps shown in this image have been extracted from one of the GNCN-t2-LΣ models trained on MNIST). Note that layers 2 and 3 (**z**^2^ and **z**^3^) control a hierarchical neuron selection pathway which leads to excitation (turning on) or suppression (turning off) of certain low-level neurons in layer 1 (**z**^1^) that contain visual features that are super-imposed to generate a final output image. The image maps shown corresponding to **z**^2^ and **z**^3^ are visualized synaptic weight vectors that appear to serve as blueprints for choosing units (white indicates neurons to excite and black indicates neurons to suppress, after normalization was applied to the original maps and values lower than a threshold of 0.5 were set to black). Red squares have been added to indicate the neurons with some of the highest activity values that select the most important visual features that will be used to compose a final output predicted image.

In Fig. 6a, we provide qualitative evidence that the latent representations of NGC (GNCN-PDH) appear to yield a stronger, natural separation of the test data points into seemingly class-respective clusters as compared to the GAN-AE (which was one of the best performing backprop-based models with respect to both log-likelihood and reconstruction error). Again, we emphasize that the GNCN-PDH acquired these representations without labeled information, meaning that the class-based relationships have emerged as a result of its very sparse neural activities. This offers some promising evidence that an NGC model’s representations offer benefits beyond the original density estimation task, allowing reuse of the same system for downstream tasks like categorization.

We hypothesize that the GNCN-t2-LΣ/GNCN-PDH’s latent codes make it easier for a linear classifier to separate out patterns by category as a result of the fact that they are sparse. Crucially, the lateral structure of these models creates columns of neural processing units that fight for the right to explain the input, meaning that only a few within a group would be active when processing particular patterns. To quantity this sparsity in the GNCN-t2-LΣ/GNCN-PDH, we measure and report in Table 3 the (mean) proportion ${\rho }_{a}^{\ell }$ of neurons at layer *ℓ* that were active (we counted whether each unit for a given pattern satisfied ${z}_{i}^{\ell } > \epsilon$, where *ϵ* = 1*e*^−6^) in each model on each dataset. We compare this to the GNCN-t1-Σ/Friston^5^, one of the best performing GNCNs that used a kurtotic prior to induce sparsity, and observe that the lateral inhibition/excitation in both the GNCN-t2-LΣ and GNCN-PDH greatly reduces the amount of neural firing required while still allowing the models to obtain top performance. Furthermore, the number of active neurons is lower in deeper layers, with 16–18% sparsity for all datasets in the top layer **z**^3^ for the GNCN-t2-LΣ. Notice that the GNCN-PDH appears to exhibit activities that are a bit less sparse than the GNCN-t2-LΣ (though still far more sparse than GNCN-t1-Σ/Friston model), although this small increased usage of neural resources appears to have contributed to the GNCN-PDH’s overall better performance in reconstruction and log-likelihood, as indicated by the previous set of experiments. We believe that the GNCN-PDH’s structured form of sparsity aids it in modeling input, i.e., yielding strong likelihood and reconstruction error, facilitating better separation of nonlinear data, and opening the door to designing models that more directly adhere to homeostatic constraints similar to those imposed by the brain. Though models like the GAN-AE, GVAE, and GVAE-CV reach good log-likelihoods too, it is possible that since their learning process focuses on shaping their latent spaces to dense, multivariate Gaussian distributions, there is a decreased chance for useful sparsity to emerge as a by-product, reducing the possibility that the latent space might result in beneficial side-effects.

**Table 3:** Latent sparsity measurements.

	GNCN-t1-Σ/Friston			GNCN-t2-LΣ			GNCN-PDH		
	${\rho }_{a}^{1}$	${\rho }_{a}^{2}$	${\rho }_{a}^{3}$	${\rho }_{a}^{1}$	${\rho }_{a}^{2}$	${\rho }_{a}^{3}$	${\rho }_{a}^{1}$	${\rho }_{a}^{2}$	${\rho }_{a}^{3}$
MNIST	0.74	0.60	0.63	0.21	0.18	0.16	0.22	0.21	0.19
KMNIST	0.35	0.50	0.67	0.24	0.21	0.18	0.26	0.24	0.21
FMNIST	0.41	0.44	0.68	0.21	0.20	0.18	0.24	0.22	0.20
CalTech	0.50	0.68	0.69	0.21	0.19	0.17	0.25	0.23	0.20

Sparsity levels in the GNCN-t1-Σ/Friston^5^ versus the GNCN-t2-LΣ and GNCN-PDH. ${\rho }_{a}^{\ell }$ indicates sparsity for layer *ℓ* in each model on each of the four databases—${\rho }_{a}^{1}$ is the measured sparsity level for the first layer of latent neural activities, ${\rho }_{a}^{2}$ is the sparsity level of the second layer, and ${\rho }_{a}^{3}$ is the sparsity level for the third layer (sparsity analysis was performed using all data samples in the training set.).

From a neurobiological perspective, it is well-known that lateral synapses often facilitate contextual processing^44^ and offer a natural form of activity sharpening^45^. We believe that this is an important structural prior that biases an NGC model, such as the GNCN-t2-LΣ or GNCN-PDH, towards acquiring more economical representations^46^, where progressively fewer neurons at layers progressively farther away from the input stimulus work to encode information (or are non-zero). Much like the more recent incarnations of spike-and-slab coding^47^, our form of structurally-enforced sparsity has a much stronger regularization effect on the model and ensures that the generative distribution of the neural activities is truly sparse. This is unlike more traditional approaches where sparsity is weakly enforced through the use of factorial kurtotic priors applied during inference^32,48^. Our NGC frameowrk not only yields naturally sparse codes but also offers flexibility in exploring other types of lateral connectivity patterns beyond the choice made in this study.

### Neural generative coding can conduct pattern completion

Another interesting ability attributed to auto-associative memory models is their ability to complete partially-corrupted or incomplete patterns. In the real world, this type of scenario would often occur in the form of object occlusion, where the view of an object might be partially obstructed from the agent’s view. Being able to imagine the rest of the object might prove useful when planning to grasp it or manipulate it in some fashion. To test each model’s ability to complete patterns, we conducted an experiment where the right half of each image in each dataset was masked and each model was tasked with predicting the deleted portions. In Table 2, we report the masked mean squared error (M-MSE, see “Methods”) of each model on each dataset’s test set.

Interestingly enough, we see that the NGC models outperform the other baselines in terms of pattern completion (with GNCN-t1-Σ/Friston and GNCN-PDH offering the most competitive performance, with respect to measured M-MSE). We furthermore provide some examples of original data patterns that were masked in Table 2b (the masked patterns appear in the first row of each dataset’s image table) contrasted with the GNCN-PDH’s completed patterns (the completed patterns appear in the second row of each dataset’s image table). We hypothesize that an NGC model’s ability to conduct pattern completion better than the autoencoder models is due to its iterative inference process.

## Discussion

Generative models based on artificial neural networks (ANNs) have yielded promising tools for estimating and sampling from complicated probability data distributions^7,8^. By re-considering how these models might operate and learn, drawing inspiration from one promising neuro-mechanistic account of how the brain interacts and adapts to its environment, i.e., predictive processing^4^, we have shown that learning a viable generative model is possible. Specifically, we propose the neural generative coding (NGC) computational framework for learning neural probabilistic models of data, implementing several concrete instantiations of NGC which we labeled as generative neural coding networks (GNCNs). In our experiments, we observe that the GNCNs are not only competitive with several powerful, modern-day backprop-based models in estimating the marginal distribution of the data but that they can generalize beyond the task they were originally trained to do. Specifically, we investigated the performance of all of these models on downstream tasks such as pattern completion and pattern categorization and discovered that the unsupervised NGC models outperformed all of the examined backprop-based baselines and were even competitive with an ANN that was directly trained to specialize for classification. As a result, for systems as complex as probabilistic generative models, we have demonstrated that crafting a more fundamentally brain-inspired approach to information processing and credit assignment can yield artificial neural systems that extract rich representations of input data in an unsupervised fashion. Our results also demonstrate, on four datasets, that even though extra computation is needed to process each input for a fixed (yet small) stimulus presentation time, the NGC models converge sooner than comparable backprop-based ones, generalizing well earlier.

To offer additional insight into what an NGC model’s intermediate representations might be doing, we examined, using the MNIST dataset, the generative synaptic weights for each layer of a trained model, of which the visualization results for the bottom layer (where **z**^1^ predicts **z**^0^) are shown in Table 4 (see Supplementary Note 8 for details of the analysis). Upon examination of these synapses, we observe that a GNCN-t2-LΣ appears to learn a latent command structure in its upper layers (layers ${{\mathfrak{N}}}^{2}$ and ${{\mathfrak{N}}}^{3}$—these appear to provide maps for turning off or on state neurons in the level below) that work in tandem to drive a dynamic composition of low-level visual features in layer ${{\mathfrak{N}}}^{1}$. In Fig. 6b, we illustrate, based on these findings, how these higher-level maps appear to interact with the layer ${{\mathfrak{N}}}^{1}$ features, ultimately composing a final output image by super-imposing an intensity-weighted set of low-level features (this final output is shown in the Output column of Table 4).

**Table 4:**
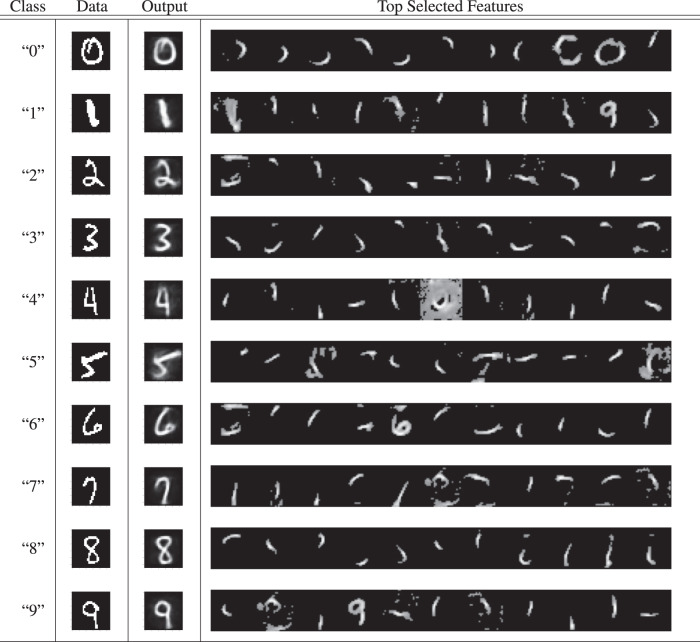
Feature composition analysis of the generative neural coding network.

The Class column shows the class label (string/identifier) of a sample (randomly selected) from MNIST, the Data column depicts the original pattern image, and the Output column presents the weighted super-position of the 30 layer 1 synaptic weight vectors (we only visually present the top 11 in the Top Selected Features column) associated with the 30 state neurons with highest activity when reconstructing the original data point. Note that the feature maps here were extracted from a GNCN-t2-LΣ trained on MNIST and were first normalized and then values lower than a threshold of 0.5 were set to black in order to improve the clearness of extracted features.

How the brain conducts credit assignment is a fundamental and open question in both (computational) neuroscience and cognitive science. There are many theories that posit how this might happen and our proposed NGC framework represents a scalable, computational instantiation of only one of them, the theory of predictive processing, suggesting that cortical regions in the brain communicate prediction errors and/or predictions across different regions through a hierarchical message passing scheme^49^. Observe that the direction that this study takes is one that starts from cognitive neuroscientific concepts and ends in the development of a statistical learning algorithm. Specifically, we have shown that one way of emulating some neurobiological principles yield agents that learn more general-purpose representations, as our results on downstream classification and pattern completion provide evidence for. As for implications for computational neuroscience itself, while an NGC model can embody concepts such as lateral competition-driven sparsity, hierarchical message passing, and local Hebbian-like synaptic adjustment, our framework lacks many important details that might allow it to serve as a means to make falsifiable claims that can be proven or disproven by neurobiological experiments. In addition, NGC models suffer from other criticisms of predictive processing in general—for example, it currently requires a one-to-one pairing of error neurons with state neurons^50^ (though this can potentially be resolved by decoupling the error neurons and introducing an extra synaptic weight matrix that connects a pool of error neurons of one size to a pool of state neurons of a different size). However, if the NGC framework is modified significantly to more faithfully model neurobiological details, e.g., synapses are constrained to take on only non-negative values and neurons communicate via spike trains (for example, the NGC could work with leaky integrate-and-fire neurons, similar to the model of ref. ^51^), it could serve as a means to facilitate refinement of predictive brain theories such as predictive processing^4^ and principles such as free energy^1^. Doing so could facilitate stronger synergy between neural computational modeling and the design of agents that solve complex tasks examined in statistical learning.

Given the difficulty in imagining how backprop, in the form it is implemented when training deep ANNs today, might occur in the brain^9,13^, there is value in not only developing approximations of it that might be more brain-like (moving machine learning a bit closer to computational neuroscience) but also in exploring alternatives that start from neuro-cognitive principles, theories, and mechanisms, creating new algorithms that embody particular ideas in cognitive neuroscience at the outset. Taking the second of the last two directions, which is in the spirit of this work and several others^52,53^, might allow us to more easily shed the constraints imposed by backprop in the effort to construct general-purpose learning agents capable of emulating more complex human cognitive function. Doing so might also allow the machine learning community to make further progress on problems even harder than generative modeling, such as the problem of learning from sparse reward signals (active inference^54,55^) and continual temporal prediction^2^.

## Methods

### Datasets

All of the datasets used in this paper, except for CalTech 101, which already contained binary images, contained gray-scale pixel feature values in the range of [0, 255]. The images in these databases were first pre-processed by normalizing the pixels to the range of [0, 1] by dividing them by 255 and finally converted to binary values by thresholding at 0.5 as in ref. ^42^.

The MNIST dataset^56^ contains 28 × 28 images containing handwritten digits across 10 categories. Fashion MNIST (FMNIST)^57^, which was proposed as a challenging drop-in replacement for MNIST, contains 28 *×* 28 gray-scale images depicting clothing items (out of 10 item classes). Kuzushiji-MNIST (KMNIST) is another 28 × 28 image dataset containing hand-drawn Japanese Kanji characters^58^. The Caltech 101 Silhouettes database^59^ contains 16 × 16 binary pixel images across 100 different categories. Each training subset had 60,000 samples and the testing subset had 10,000 (the standard test split of each dataset was used in this paper), with the exception of Caltech 101, which had 8596 training samples and 2302 test samples. A validation subset of 2000 samples was drawn from each training set to be used for tuning model meta-parameters (the Caltech validation subset had 2257 samples).

### Baseline model descriptions

The baseline backprop-based models implemented for this article included a regularized auto-associative (autoencoding) network (RAE), a Gaussian variational autoencoder with fixed (spherical) variance (GVAE-CV)^60^, a Gaussian variational autoencoder (GVAE)^7^, and an adversarial autoencoder (GAN-AE)^61^. All models were constrained to have four layers like the NGC models. For all backprop-based models, the sizes of the layers in between the latent variable and the input layer ${{{{{{{{\bf{z}}}}}}}}}^{0}$ were chosen such that the total synaptic weight count of the model was (at most) approximately equal to the GNCNs (autoencoder layer size was tuned on validation data), the linear rectifier was used for the internal activation function, i.e., *ϕ*^*ℓ*^(*v*) = *max*(0, *v*), and weight values (for the encoder and decoder) were initialized from a centered Gaussian distribution with a standard deviation *σ* that was tuned on held-out validation data. To further improve generalization ability, the decoder weights of all autoencoders were regularized and some autoencoder models had specific meta-parameters that were tuned using validation data. Notably, the GAN-AE was the only model that required a specialized gradient descent rule, i.e., Adam^62^, in order to obtain good log-likelihood and to stabilize training (stability is a known issue related to GANs^8^). Finally, we implemented an optimized Gaussian mixture model (GMM) baseline model that was fit to the training data via expectation-maximization with the number of mixture components chosen based on preliminary experiments that yielded best performance. The only GMM implementation detail worthy of note was that we clipped the images sampled from the mixture model to lie in the range [0, 1] (which improved likelihood slightly).

### Experimental setup and task design

#### Training setup

The parameters of all models, whether they were updated via backprop or by the NGC learning process, were all optimized using stochastic gradient descent using mini-batches (or subsets of data, randomly sampled without replacement) of 200 samples for 50 passes through the data (epochs). For the backprop-based models, we re-scaled the gradients^19^ by re-projecting them to a Gaussian ball with radius of 5.0, which we found ensured stable performance across trials. For each model, upon completion of training, we fit a Gaussian mixture model (GMM) to the topmost neural activity layer (to serve as the model prior). This density estimator was trained with expectation-maximization and contained *K* = 75 components, where each component *k* ∈ *K* defines a multivariate Gaussian with mean *μ*_*k*_ and covariance Σ_*k*_ parameters as well as a mixing coefficient *π*_*k*_.

#### The density modeling task

Given a dataset **X** ∈ {0, 1}^*D*×*S*^, where *S* is the number of vector pattern samples and *D* is the dimensionality of any given pattern, the goal is to learn a density model of *p*(**X**), or the probability of sensory input data, where a subset of *B* vectors is denoted as **x** ∈ {0, 1}^*D*×*B*^. We parameterize the probability distribution *p*(**X**) via *p*_Θ_(**X**) by introducing learnable parameters Θ. Since computing the marginal log-likelihood $\log {p}_{{{\Theta }}}({{{{{{{\bf{X}}}}}}}})$ directly is intractable for all the models in this paper, we estimate it by calculating a Monte Carlo estimate using 5000 samples according to the recipe: (1) sample the GMM prior, (2) ancestrally sample the directed neural generative model (whether it is a baseline or a GNCN) given the samples of the prior.

The reconstruction metric, binary cross-entropy (BCE), also known as the negative Bernoulli log-likelihood, was computed as follows: ${{{{{\rm{BCE}}}}}}({{{{{{{\bf{X}}}}}}}},\hat{{{{{{{{\bf{X}}}}}}}}})=-\frac{1}{S}\mathop{\sum }\nolimits_{s = 1}^{S}\mathop{\sum }\nolimits_{d = 1}^{D}\left({{{{{{{{\bf{X}}}}}}}}}_{ds}\log ({\hat{{{{{{{{\bf{X}}}}}}}}}}_{ds})+(1-{{{{{{{{\bf{X}}}}}}}}}_{ds})\log (1-{\hat{{{{{{{{\bf{X}}}}}}}}}}_{ds})\right)$ (measured in nats). $\hat{{{{{{{{\bf{X}}}}}}}}}\in {\{0,1\}}^{D\times S}$ is the predicted sensory input (matrix) produced by a model under evaluation.

#### The pattern completion task

For this task, we test each model’s ability to complete patterns where the images of each dataset were partially masked and each model was tasked with completing the masked images. Specifically, half of each image **x** (containing $\sqrt{D}$ columns of $\sqrt{D}$ pixels) was masked according to a binary mask **m** where $\sqrt{D}/2$ columns were set to 1 and the rest to 0, i.e., **x**_*m*_ = **x** ⊙ **m**. We report the masked mean squared error (M-MSE) of each model on each dataset’s test set, which is computed per image as follows: $\,{{\mbox{M-MSE}}}\,({{{{{{{\bf{X}}}}}}}},\hat{{{{{{{{\bf{X}}}}}}}}},{{{{{{{\bf{M}}}}}}}})={((\hat{{{{{{{{\bf{X}}}}}}}}}-{{{{{{{\bf{X}}}}}}}})\odot (1-{{{{{{{\bf{M}}}}}}}}))}^{T}\cdot ((\hat{{{{{{{{\bf{X}}}}}}}}}-{{{{{{{\bf{X}}}}}}}})\odot (1-{{{{{{{\bf{M}}}}}}}}))$ (measured in nats), where **M** ∈ {0, 1}^*D*×*S*^ is a masking matrix where each column contains one mask vector **m** per image vector **x**.

#### The classification task

In this task, each model’s prediction error is measured on the test sample’s label set. This is possible given that each dataset **X** also comes with a set of target annotations that we encode into a *C*-dimesnional space, yielding the binary label matrix **Y** ∈ {0, 1}^*C*×*D*^ (one label vector per sample), where *C* is the number of unique categories labeled a priori. We measure the classification error (**Err**) (as a percentage %) on the test set, as follows:

$$\,{{\mbox{Err}}}\,({{{{{{{\bf{Y}}}}}}}},\hat{{{{{{{{\bf{Y}}}}}}}}})=\left(1-\frac{1}{D}\,\mathop{\sum }\limits_{d=1}^{D}\left\{\begin{array}{ll}1&{\hat{y}}_{d}={y}_{d}\\ 0&{\hat{y}}_{d}\ne {y}_{d}\end{array}\right.\right)* 100$$

where, for the *d*th data sample, ${\hat{y}}_{d}=\arg \mathop{\max }\limits_{c}({\hat{{{{{{{{\bf{Y}}}}}}}}}}_{:,d})$ is the class index chosen by the model and ${y}_{d}=\arg \mathop{\max }\limits_{c}({{{{{{{{\bf{Y}}}}}}}}}_{:,d})$ is the index of the actual class label. The subscript symbol :,*d* indicates that we extract the *d*th row from a matrix, e.g., **Y**. Note that $\hat{{{{{{{{\bf{Y}}}}}}}}}\in {{{{{{{{\mathcal{R}}}}}}}}}^{C\times D}$ is the collected set of predictions from the linear classifier fit to a model’s latent space. The final score is multiplied by 100 to obtain a percentage value.

### Neural generative coding model procedures

In this section, we detail the sampling and image completion procedures for the GNCN models.

#### Sampling from the model

Since the optimization procedure, whether via gradient descent or another method, is not guaranteed to find globally optimal parameter settings (since the objective function is not convex), the distribution of the latent state **z**^*L*^ of the final layer will not be Gaussian. To estimate it, after training on the data, we obtain the corresponding ${{{{{{{{\bf{z}}}}}}}}}_{i}^{L}$ value for each data point **x**_*i*_. We fit a Gaussian mixture model to this collection of values ${{{{{{{{\bf{z}}}}}}}}}_{1}^{L},\ldots ,{{{{{{{{\bf{z}}}}}}}}}_{D}^{L}$ (where *D* is the number of training points). Then, in order to reduce variance during sampling, we take the following approach. First, we sample **z**^*L*^ from the Gaussian mixture model, recursively set ${\overline{{{{{{{{\bf{z}}}}}}}}}}^{\ell -1}\leftarrow {g}^{\ell -1}\left({{{{{{{{\bf{W}}}}}}}}}^{\ell }\cdot {\phi }^{\ell }({{{{{{{{\bf{z}}}}}}}}}^{\ell })+{\alpha }_{m}({{{{{{{{\bf{M}}}}}}}}}^{\ell +1}\cdot {\phi }^{\ell +1}({{{{{{{{\bf{z}}}}}}}}}^{\ell +1}))\right)$ and output **z**^0^. This is similar to how variational auto-encoders are sampled in practice, where the input is a Gaussian and the output is the mean vector.

#### Image completion

In the event that an incomplete sensory input **x** is provided to an NGC model, i.e., portions of **x** are masked out by the variable ${{{{{{{\bf{m}}}}}}}}\in {\{0,1\}}^{{J}_{0}\times 1}$, we may infer the remaining portions of **x** by utilizing the output error neurons of any GNCN and treating its bottom sensory layer **z**^0^ as a partial latent state. Specifically, we update the missing portions, i.e., 1−**m**, of **z**^0^ as follows:

19 $${{{{{{{{\bf{z}}}}}}}}}^{0}={{{{{{{\bf{x}}}}}}}}\odot {{{{{{{\bf{m}}}}}}}}+\left({{{{{{{{\bf{z}}}}}}}}}^{0}+\beta (-\frac{\partial \psi }{\partial {\overline{{{{{{{{\bf{z}}}}}}}}}}^{0}})\right)\odot (1-{{{{{{{\bf{m}}}}}}}})={{{{{{{\bf{x}}}}}}}}\odot {{{{{{{\bf{m}}}}}}}}+\left({{{{{{{{\bf{z}}}}}}}}}^{0}-\beta {{{{{{{{\bf{e}}}}}}}}}^{0}\right)\odot (1-{{{{{{{\bf{m}}}}}}}}).$$

### Programming

The experiments, the baseline models, and the proposed computational framework were written in Python, using the Numpy and TensorFlow 2 libraries. Experiments were sped up using a GPU card.

## Supplementary information

Supplementary Information

## Data Availability

The datasets, i.e., MNIST^56^, Fashion MNIST^57^, NotMNIST^63^, and Caltech 101 Silhouettes^59^, used by this study are publicly available.

## Code availability

All code used for the training and analysis of the NGC models is available at the link: https://github.com/ago109/ngc-learn.
