# Supplementary material for: The neural coding framework for learning generative models
Source: Nat Commun. 2022 Apr 19;13:2064. doi: 10.1038/s41467-022-29632-7 (PMC9018730; doi:10.1038/s41467-022-29632-7)
Supplement: Supplementary file 1 — Supplementary Information [file 41467_2022_29632_MOESM1_ESM.pdf]

SUPPLEMENTARY MATERIAL: THE NEURAL CODING FRAMEWORK FOR LEARNING GENERATIVE MODELS

Alexander Ororbia*
Department of Computer Science
Rochester Institute of Technology
Rochester, NY 14623
ago@cs.rit.edu

Daniel Kifer
Department of Computer Science & Engineering
The Pennsylvania State University
State College, PA 16801
duk17@psu.edu

Supplementary Note 1: On The Neural Generative Coding Framework, Its Naming Scheme, and Its Generality

NGC Model Structure and Naming Convention: Naming GNCN models under the NGC framework entails appending a suffix to the end of the model sub-name. The hyphenated suffix "-t1" refers to a GNCN model that uses "Type 1" error synapses, or, specifically, (virtual) error synapses that are a function of the GNCN's forward generative weights, i.e., they are not physically separate/distinct synapses. The suffix "-t2" refers to a GNCN model with "Type 2" error synapses, or separate learnable synaptic parameters that focus on solely transmitting error messages throughout the model. An additional suffix is added to the model name depending on whether on it contains lateral synapses in its state variables, i.e., "-L", and whether or not it contains lateral precision weights in its error neurons, i.e, "-Σ". As a complete example, a GNCN that contains Type 2 error synapses and lateral synapses in both its state and error neurons would be designated as GNCN-t2-LΣ. The table immediately below presents the four key models and their names investigated in this paper, with a further breakdown of their key properties in the additional columns ("Prec Wghts" stands for precision weights, "Lat Wghts" stands for lateral weights, and the last two columns depict whether non-hierarchical synapses are used, i.e., $\alpha_m = 1$, and whether the partial derivatives of the model's activation functions, i.e., $\partial\phi$, are included in its state update equation).

**NGC Model Name**	Error Type	Prec Wghts	Lat Wghts	α_m Value	Uses $\partial\phi$
GNCN-t1/Rao	Type 1	No	No	0	Yes
GNCN-t1-Σ/Friston	Type 1	Yes	No	0	Yes
GNCN-t2-LΣ	Type 2	Yes	Yes	0	No
GNCN-t2-LΣ-PDH (GNCN-PDH)	Type 2	Yes	Yes	1	No

While this study explores four variant GNCN models that can be derived from the general NGC computational framework proposed in this study, there are many other possible architectures that can handle other styles of problems. This flexibility is owed to the fact that our NGC framework supports asymmetry with respect to the structure of the forward generative pathway and that of the error transmission pathway. Notably, if an NGC model utilizes a non-hierarchical structure in its generative/prediction neural structure ($\alpha_m = 1$), as in the case of one of this paper's main models, it receives one final suffix "-PDH" (for partially decomposeable hierarchy). Note that in the case of the GNCN-t2-LΣ-PHD, we, in this paper, for further convenience, abbreviate it to GNCN-PDH. Alternatively, if an NGC model contains a non-hierarchical error structure (given that is forward and error transmission pathways are not strictly required

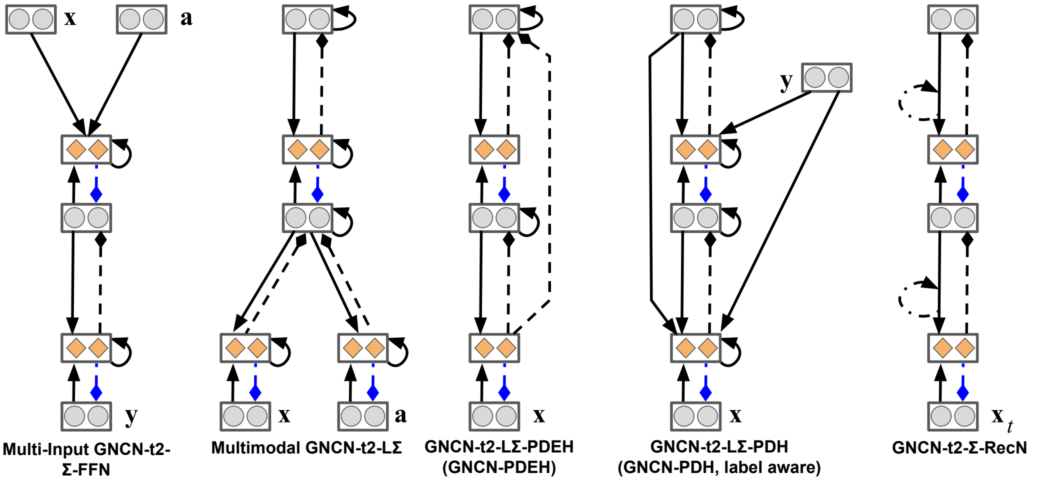

Supplementary Figure 1: **Variant NGC Architectures:** Ordered left to right, possible architectures in NGC include: 1) a multi-input GNCN-t2-Σ, 2) a multimodal GNCN-t2-LΣ, 3) a GNCN-t2-L with an asymmetric error transmission pathway (a GNCN-PDEH), 4) a label-aware GNCN-PDH (or a multi-input GNCN-t2-LΣ) with an asymmetric generative pathway, and 5) a recurrent GNCN-t2-LΣ (temporal NGC).

to be symmetric), it would receive the final suffix "-PDEH" (for partially decomposeable error hierarchy). Note that certain special cases of our framework have been further provided special name designations to highlight their source studies, e.g., GNCN-t1 is also referred to as GNCN-t1/Rao [40] and GNCN-t1-Σ is also referred to as GNCN-t1-Σ/Friston [13].

Figure 1 depicts five other alternative neural circuit structures that would tackle, respectively, 1) clamped, multiple input to generated/predicted outputs (as in the case of tasks such as direct classification), 2) multi-modal generative modeling (for example, crafting a circuit that jointly learns to synthesize an image and discrete one-hot encoding of a word/character at time step t within a sequence), 3) a generative model where upper layers receive error messages from layers other than its immediately connected one (a GNCN-PDEH, where PDEH means partially decomposable error hierarchy), i.e., layer $\ell = 2$ receives error messages from layer $\ell = 1$ and $\ell = 0$, 4) a label-aware generative model that forms a partially decomposable hierarchy in its forward generative structure (GNCN-PDH driven by labels as input), and 5) one type of temporal/recurrent NGC, where the predictive outputs of each state region are further conditioned on their previous values, i.e., $\bar{\mathbf{z}}^\ell_t$ would be function of the state $\mathbf{z}^{\ell+1}$ and $\bar{\mathbf{z}}^\ell_{t-1}$ (as indicated by the curled dash-dotted arrows), since the input takes on temporal/ordered form (as in frames of a video), i.e., $\mathbf{x}_t$ where t marks a step in time.

If more complex transformations are needed to map a state layer's activity $\mathbf{z}^\ell$ to a set of nonlinear prediction values $\bar{\mathbf{z}}^\ell$, for example using multiple self-attention heads as in the case of transformer networks [8], one could opt to leverage back-propagation to locally compute the weight adjustments to the attention head synapses utilizing NGC's online objective is total discrepancy, which is an approximate form of (variational) free energy [35].

Finally, in Table 1, we present a small nearest neighbor analysis of the samples generated by two NGC models – GNCN-t1-Σ (GNCN-t1-Σ/Friston) and GNCN-t2-LΣ – and backprop-based autoencoder models – a regularized autoencoder (RAE) and an adversarial autoencoder (GAN-AE). Specifically, we forced each model, after training on each database, to synthesize a pool of data samples equal in size to the original dataset, e.g., for MNIST, each model created $58,000$ samples (since 2000 were used for validation). Then, we randomly sampled one original data point from each class of each database and performed a nearest neighbor search (using the Euclidean distance function) to select out of the synthesized patterns, for each model, the single fantasy with the lowest distance measurement, i.e., the single top nearest neighbor. We present the visual results of this simple analysis in Table 1 with the top row displaying the ground truth

Model	MNIST Samples	KMNIST Samples
Truth	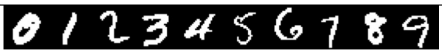	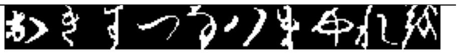
RAE	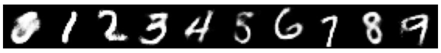	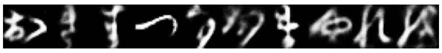
GAN-AE	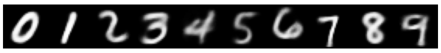	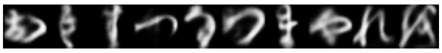
GNCN-t1-Σ	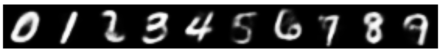	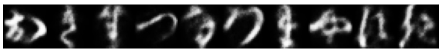
GNCN-t2-LΣ	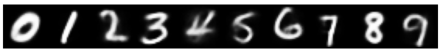	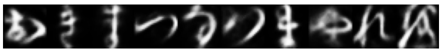
	**FMNIST Samples**	**CalTech Samples**
Truth	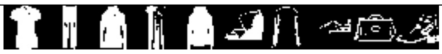	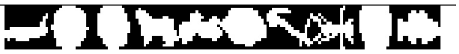
RAE	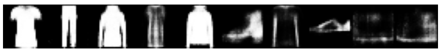	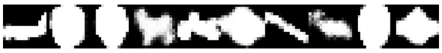
GAN-AE	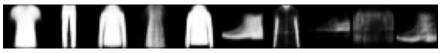	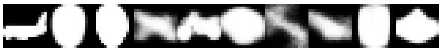
GNCN-t1-Σ	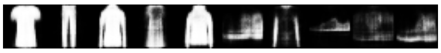	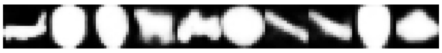
GNCN-t2-LΣ	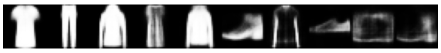	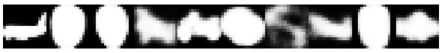

Supplementary Table 1: **Nearest neighbor sample matches:** For each dataset, i.e., MNIST, KMNIST, FMNIST, and CalTech, we depict the nearest neighbor sample for each class from: ground truth (Truth), the regularized autoencoder (RAE), the adversarial autoencoder (GAN-AE), the GNCN-t1-Σ (also referred to as GNCN-t1-Σ/Friston), and the GNCN-t2-LΣ. Specifically, we present the top one nearest neighbor match to a randomly sampled digit for each class from the original dataset.

pattern (the Truth row) and each column representing a different class. Each row below the Truth row presents the nearest neighbor fantasies of each model.

Supplementary Note 2: Related Work on Biologically-Inspired Models and Learning Algorithms

As mentioned in the introduction, some of the more notable criticisms of backprop include:

1. Synapses that make up the forward information pathway need to directly be used in reverse to communicate teaching signals (the weight transport problem),
2. Neurons need to be able to communicate their own activation function's first derivative,
3. Neurons must wait for the neurons ahead of them to percolate their error signals way back before adjusting their own synapses (the update-locking problem),
4. There is a distinct form of information propagation through a long, global error feedback pathway that only affect weights but does not (at least directly) affect the network's internal representations,
5. The error signals have a one-to-one correspondence with neurons.

The properties above are inherent to backprop and do not conform to known biological feedback mechanisms underlying neural communication in the brain [7]. The brain, in contrast, is heavily recurrently connected [2, 9], allowing for complementary pathways to form that would allow for percolation of error/mismatch information [28, 44]. Biological neurons communicate binary spike signals, making it unlikely that they also sport specialized circuitry to communicate the derivative of a loss function with respect to their activities [18] (though some recent studies have suggested that real neurons might communicate with rate codes [27]). Furthermore, it is more commonly accepted that neurons in the brain learn "locally" [16, 11], modulated globally by signals provided through neuromodulators such as dopamine, i.e., they operate with only immediately available information (such as their own activity and that of nearby neurons that they are connected to), making it unlikely that a global feedback pathway drives synaptic adjustments.

In addition, several of the problems above result in practical problems – the weight transport problem has been shown to create memory access pattern issues in hardware implementations [6] and the global feedback pathway itself is a key source behind the well-known exploding and vanishing gradient problems [38] in deep ANNs, yielding unstable/ineffective learning unless specific heuristics are employed.

In recent research, developing learning procedures that enable backprop-level learning while embodying elements of actual neuronal function has seen increasing interest in the machine learning community. However, while insights provided by each development have proven valuable, increasing the evidence that shows how a backprop-free form of adaptation can be consistent with some aspects real networks of neurons, many of these ideas only address one or a few of the issues described earlier. Random feedback alignment algorithms [3, 25] address the weight transport problem, and to varying degrees, the update locking problem [32, 30, 12], but are fundamentally emulating backprop's differentiable global feedback pathway to create teaching signals (and pay a reduction in generalization ability the farther away they deviate from backprop [4, 12]). In addition, experimentally, the success of these approaches depends on how well the feedback weights are chosen a prior (instead of learning them). Other procedures, like local representation alignment [36] and target propagation [20, 24], which also resolve the weight transport problem and eshew the need for differentiable activations, fail to address the update locking problem, since the various incarnations of these require a full forward pass to initiate inference. Procedures have been proposed to address the update locking problem, such as the method of synthetic gradients [22], but still require backprop to compute local gradients. It remains to be seen how these algorithms could be adapted to learn generative models. Other systems, such as those related to contrastive Hebbian learning (CHL) [31, 34, 43], are much more biologically-plausible but often require symmetry between the forward and backward synaptic pathways, i.e., failing to address weight transport. More importantly, CHL requires long settling phases in order to compute activities and teaching signals, resulting in long computational simulation times. However, while some of these algorithms have shown some success in ANN training [24, 25, 36], they focus on classification, which is purely supervised and arguably a simpler problem than generative modeling.

Boltzmann machines [1], which are generalizations of Hopfield networks [26, 21] to incorporate latent variables, are a type of generative network that also contains lateral connections between neurons much as the models in our NGC framework do. While training for the original model was slow, a simplification was later made to omit lateral synapses, yielding a bipartite graphical model referred to as a harmonium, trained by contrastive divergence [17], a local contrastive Hebbian learning rule. However, while powerful, the harmonium could only synthesize reasonable-looking samples with many iterations of block Gibbs sampling and the training algorithm suffered from mixing problems (leading low sample diversity among other issues) [10]. Another kind of generative model [19] can be trained with the wake-sleep algorithm (or the up-down algorithm in the case of deep belief networks [19]), where an inference (upward) network and a generative (downward) network are jointly trained to invert each other. Unfortunately, wake-sleep suffers from instability, struggling to produce good samples of data most of the time, due to the difficulty both networks have in inverting each other due to layer-wise distributional shift. Motivated by the deficiencies in models learned by contrastive divergence/wake-sleep, algorithms have been created for auto-encoder-based models [5] but most efforts today rely on backprop.

Supplementary Note 3: On Evaluating Mode Capture

Given that a common problem in training generative models is mode collapse, we measure some distributional properties of several of our baseline models and NGC models. Note that it is difficult to directly and automatically determine the labels of the samples produced by the unsupervised models investigated in this paper (aside from manual qualitative inspection) and it is an open research area/problem to develop better measurements for evaluating mode-capturing ability of generative models in general. Among the myrid of current experimental approaches proposed for measuring the degree of modal capture, we opted to implement and measure the number of statistically different bins (NBD) from [41], which has been argued to be a potentially useful metric for evaluating the degree of mode collapse that a given generative model might have experienced (values closer to 0 mean that the model has likely captured most of the modes

Model		G-KL	NDB		G-KL	NDB
Baseline	MNIST	1229.3871	1.00 ± 0.0	KMNIST	1988.3081	1.00 ± 0.0
RAE		180.621 ± 5.966	0.72 ± 0.014		668.821 ± 36.12	0.72 ± 0.014
GVAE-CV		254.976 ± 3.036	0.63 ± 0.042		704.495 ± 29.394	0.645 ± 0.049
GVAE		295.973 ± 23.266	0.71 ± 0.028		804.943 ± 18.041	0.685 ± 0.021
GAN-AE		540.408 ± 25.943	0.665 ± 0.035		1325.641 ± 39.115	0.74 ± 0.014
GNCN-t1/Rao		682.454 ± 1.732	0.77 ± 0.042		1316.362 ± 2.463	0.755 ± 0.049
GNCN-t1-Σ/Friston		531.206 ± 3.735	0.775 ± 0.021		1205.361 ± 6.5	0.79 ± 0.014
GNCN-t2-LΣ		596.67 ± 0.106	0.78 ± 0.014		1218.252 ± 2.761	0.795 ± 0.12
GNCN-PDH		446.053 ± 3.369	0.69 ± 0.002		1150.434 ± 2.68	0.745 ± 0.007
Baseline	FMNIST	1811.2068	1.0 ± 0.0	CalTech	704.3340	1.0 ± 0.0
RAE		551.805 ± 14.6	0.74 ± 0.028		57.235 ± 4.925	0.34 ± 0.042
GVAE-CV		661.615 ± 4.182	0.725 ± 0.007		216.271 ± 8.387	0.32 ± 0.014
GVAE		797.931 ± 1.731	0.685 ± 0.021		205.223 ± 1.316	0.27 ± 0.002
GAN-AE		1279.573 ± 43.131	0.805 ± 0.035		266.874 ± 8.692	0.365 ± 0.092
GNCN-t1/Rao		1173.705 ± 8.71	0.79 ± 0.014		314.684 ± 1.871	0.325 ± 0.021
GNCN-t1-Σ/Friston		1040.528 ± 1.019	0.805 ± 0.021		300.303 ± 0.659	0.4 ± 0.028
GNCN-t2-LΣ		1180.764 ± 7.35	0.86 ± 0.002		290.617 ± 0.299	0.395 ± 0.049
GNCN-PDH		1091.876 ± 8.8	0.76 ± 0.014		204.032 ± 1.067	0.31 ± 0.099

Supplementary Table 2: **Distributional measurements:** Model ability to match the implicit modes underlying each dataset, i.e., MNIST, KMNIST, FMNIST, and CalTech. Metrics reported include the Gaussian Kullback-Leibler divergence (G-KL) and number of statistically different bins (NDB). (Metrics averaged over 10 trials - we report their mean and standard deviation.)

of the data's underlying distribution). To complement this metric, we also measure the Kullback-Leibler divergence (KL-D) between a pool of samples generated by our model (equal to the size of the original dataset) and the original dataset samples – we estimate the empirical mean and covariance matrices of each and calculate the closed-form multivariate Gaussian KL-D, to be specific.

To further utilize the labels, we also present results using another approach proposed in [42]. Specifically, we train a well-regularized multilayer perceptron (MLP) classifier (as mentioned above) on the full original dataset and use it to automatically annotate the samples produced by a given generative model. Once the samples have been annotated, we compute the frequencies of each label class (and furthermore, normalize these values to lie in the range $[0, 1]$) and plot these class probabilities in Figure 2 (we also plot the ground truth distribution for reference). Also note that, since even a powerful MLP classifier such as the one we trained incurs error (it naturally cannot reach 100% test error), the frequency measurements should be taken with annotator error in mind.

Based on the results presented in Figure 2 and Table 2, we see that while it does not appear that the predictive processing NGC models suffer from any severe form of mode collapse, they do not appear to capture the frequency distribution of the classes as well as the GAN-AE. With respect to the G-KL and NBD metrics, it does appear that the GNCN-PDH and GNCN-t1-Σ do well (often performing among the top scores across all datasets), although the GAN-AE and GVAE perform the best currently with respect to these mode measurements. Future work will entail uncovering the potential reason why the NGC models do not, at least upon first examination, match the class frequency of the data as well as the autoencoders.

Supplementary Note 4: On Model Complexity of Neural Generative Coding

On Setting Parameter Complexity

Model complexity was selected based on consideration of the hardware resources available and preliminary experimentation with the validation set of each dataset, i.e., each benchmark had a validation subset that was randomly sampled without replacement (per class) from the training set, as described in the

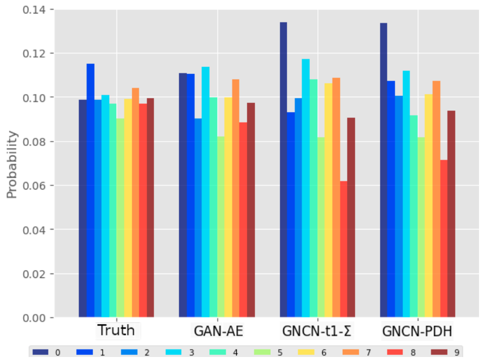

(a) MNIST.

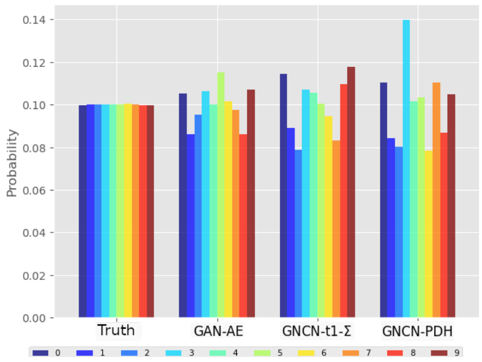

(b) KMNIST.

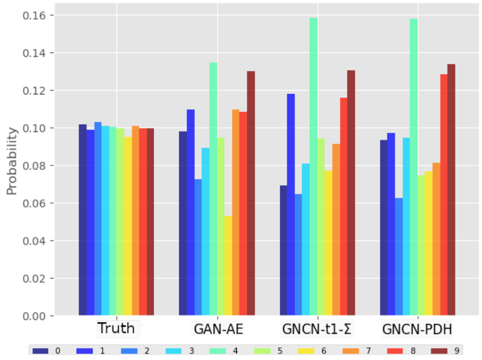

(c) FMNIST.

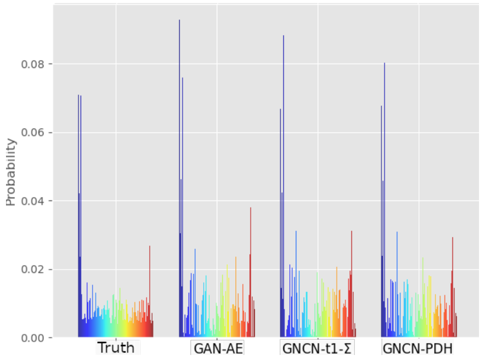

(d) CalTech.

Supplementary Figure 2: **Class distribution visualization:** Approximate label distributions (averaged over 10 trials) produced by each model – samples from each model were automatically annotated by a regularized MLP discriminator separately trained on original ground truth data. (a) MNIST class distribution, (b) KMNIST class distribution, (c) FMNIST class distribution, and (d) CalTech class distribution. (Note that GNCN-t1-Σ is also referred to as GNCN-t1-Σ/Friston.)

paper. Backprop-based variational autoencoders are typically designed with low-dimensional latent spaces in mind so we investigated different latent code sizes in the range of $[5, 30]$ and found that 20 worked best.Then, we constrained the GNCNs that had lateral synapses to only have 20 neural columns (since each column roughly functions as a latent variable) in their topmost layers and explored values for the lower levels in the range of $[100, 400]$ and found that 360 was a good choice (GNCN-t1 [40] and GNCN-t1-Σ [13] were set to use 360 neurons in the top layer). For the autoencoder models, the hidden layer sizes were set to be equal and a coarse grid search was conducted through the range between 100 and the maximum number possible for equal-sized layers such that the model could only have a maximum number of parameters equal to the total of the NGC models (meaning they could have fewer synapses if that yielded better performance on the validation set), to ensure fair comparison.

The optimal dimensionality of $\mathbf{z}^\ell$ was also tuned through preliminary experimentation using held-out validation data. We chose the linear rectifier activation function for the NGC models because we desired strictly positive activity values (which work well with the formulation of lateral inhibition we present in this work). For the GNCN-t1-Σ [13], we found that the linear rectifier worked best while the hyperbolic tangent worked best for GNCN-t1 [40] (for both of these models, the coefficient weighting the kurtotic prior was tuned on validation data).

On Model Run-time Complexity

Note that an NGC model, require multiple steps of processing to obtain their latent activities for a given input. Naturally, per sample, this means that all of the predictive processing models we have explored would be slower than the feedforward autoencoder models (which conduct inference with a single feedforward pass). In short, while an autoencoder (with $L+1$ layers) would roughly only require $2*L$ matrix multiplications (the most expensive operation in the neural systems we investigate), any NGC predictive processing model would require at least $2*L*T$ multiplications. Note that, as observed in our data efficiency plots in the main paper, we note that this more expensive per-sample cost is desirably offset by convergence with fewer samples in comparison to backprop models. Furthermore, specialized hardware could take advantage of the NGC's inherent parallelism to speed up the process.

One key to reducing the inherent cost of an NGC model's iterative processing include designing alternative state update equations (whereas the ones explored in this paper embody a form of Euler integration, one could design higher-order integration steps, such as those based on the midpoint method or Runga-Kutta). Another solution could be to craft an amortized inference process, where another neural model (much akin to the encoder in a variational autoencoder) learns to infer the value of the state variables at the end of an expensive iterative inference process so as to ultimately reduce the amount of processing steps per sample required. We leave investigation of these remedies for future work.

Supplementary Note 5: On the Omission of Activation Derivatives

Removing the activation derivative as we did in our GNCN-t2-LΣ and GNCN-PDH models could be argued to lead to possible value fluctuations that lead to unstable dynamics. Nonetheless, experimentally, we did not observe any strong weight fluctuations in our simulations and we believe that such fluctuations (much like the growing weight value problem inherent to many Hebbian update rules) are unlikely given that our model weight (columns) are constrained to have unit norms. Furthermore, the step size β is usually kept within the range of $[0.02, 0.1]$ and the leak variable $-\gamma \mathbf{z}^\ell$ helps to smooth out the values and prevent the occurrence of large values/magnitudes (serving as sort of an L2 penalty over the latent state activities).

In [37, 36], it was argued that so long as the activation function was monotonically increasing (with a similar condition imposed for stochastic activation functions), then the learning process would be stable and the benefit that the point-wise derivative offered would be absorbed by the error synaptic weights introduced to carry error signals. However, note that an approximation for the derivative in the form of a prefactor (derivative) term could be designed/introduced to further safeguard against potential fluctuations (and this will be the subject of future work).

Supplementary Note 6: Generating the Lateral Competition Matrices

In the main paper, we introduced a lateral competition matrix $\mathbf{V}^\ell$ that directly affects the latent state $\mathbf{z}^\ell$. It is created to contain the self-excitation weights and lateral inhibition weights by using the following matrix equation: $\mathbf{V}^\ell = \alpha_h(\mathbf{M}^\ell) \odot (1-I) - \alpha_e(I)$, where I is the identity matrix and the masking matrix $\mathbf{M}^\ell \in \{0,1\}^{J_\ell \times J_\ell}$ is set by the experimenter (placing ones in the slots where it is desired for neuron pairs to laterally inhibit one another). In this study, we set $\alpha_e = 0.13$ (the self-excitation strength) and $\alpha_h = 0.125$ (the lateral inhibition strength). Our mask matrix M^ℓ, which emphasized a type of group or neural-column form of competition, was generated by the following process:

1. create J_ℓ/K matrices of shape $J_\ell \times K$ of zeros, i.e., $\{\mathbf{S}_1, \mathbf{S}_2, \cdots, \mathbf{S}_k, \cdots, \mathbf{S}_C\}$ (where $C = J_\ell/K$)
2. in each matrix $\mathbf{S}_k$ insert ones at all combinations of coordinates $c = \{1, \cdots, k, \cdots, K\}$ (column index) and $r = \{1 + K*(k-1), \cdots, k + K*(k-1), \cdots, K + K*(k-1)\}$ (row index)
3. concatenate the J_ℓ/K matrices along the horizontal axis, i.e., $\mathbf{M}^\ell =< \mathbf{S}_1, \mathbf{S}_2, \cdots, \mathbf{S}_C >$.

Note that for our proposed integration of the latetal synapses in the state neuron layers, we start out with a probabilistic model and then modify it by introducing sparsity driven by lateral synaptic weights (a neuroscience-inspired idea), which directly modify the values of the state neurons (serving as sort of filter). While we cannot justify them in the probabilistic model, our experiments in the Results section of the main paper show that they improve over those that do not employ them, such as GNCN-t1-Σ [13] and GNCN-t1 [40] (which both impose a Laplace distribution over their state neurons to encourage sparsity). Part of our future work will be to derive a probabilistic interpretation of our particular extensions to the NGC model.

With respect to backprop-based neural systems, one could also introduce stateful neurons with similar connectivity to our lateral and precision synapses above by introducing recurrence (as is done in recurrent neural networks). However, to update the weight parameters, one would have to resort to backprop through time (BPTT) and unroll the model over T steps in time. This would require creating a very deep computational graph and storing the activities and gradients at each time step before a final update to each synaptic weight matrix could be calculated, creating a very expensive memory footprint. An NGC model, in contrast, does not require unrolling and the large memory footprint associated with BPTT-trained recurrent networks.

Supplementary Note 7: Autoencoder Baseline Model Descriptions

To make learning the decoder function (NN) described in the main paper tractable, it is common practice in the deep learning literature to introduce a supporting function known as the *encoder* [23]. The encoder (NN_e), parameterized by a feedforward network, takes in the input stimulus $\mathbf{x}$ and maps it to $\mathbf{z}$ or to a distribution over $\mathbf{z}$. Depending on the choice of encoder, one can recover one of the four main baselines we experimented with in this paper.

For all backprop-based baseline models in this paper, the decoder of each was regularized with an additional L2 penalty. Specifically, this meant that their data log likelihood objectives always took the form: $\psi_{reg} = \psi + \Omega(\Theta_{\text{NN}})$, where $\Theta_{NN} = \{\mathbf{W}^L, \cdots, \mathbf{W}^\ell, \cdots, \mathbf{W}^1\}$ contains all of the weight matrix parameters of the decoder NN. Ω_{NN} is the regularization function applied to the decoder, i.e., $\Omega_{\text{NN}} = -\lambda \sum_{\mathbf{W}^\ell \in \Theta} ||\mathbf{W}^\ell||_2^2$ where $||\mathbf{W}^\ell||_2$ denotes computing the Frobenius norm of $\mathbf{W}^\ell$. During training/optimization with gradient ascent, we do not constrain the column norms of any of the weight matrices for any of the baseline models (as we do for the GNCNs) as we found that doing so worsened their generalization ability.

Furthermore, the number of total layers in the decoder for any model was set to be four – one output and one input layer with two hidden layers in between. The encoder was constrained to be the same – one input and one output layer with layers in between (in the case of the GVAE, GVAE-CV, and GAN-AE, the encoder's output is technically split into two blocks, as described later). The sizes of the hidden layers were set such that the total number of learnable model weights were approximately equal across all baselines and GNCNs (maximum was $1,400,000$ synapses), which means that all models were forced to have the same parameter complexity to avoid any unfair advantages that might come from over-parameterization.

Regularized Auto-encoder (RAE): The encoder NN_e is designed to be a feedforward network of L layers of neurons. Each layer is a nonlinear transformation of the one before it, where $\hat{\mathbf{z}}^\ell = \phi^\ell(\mathbf{E}^\ell \cdot \hat{\mathbf{z}}^{\ell-1})$. Like in the decoder, ϕ^ℓ is an activation function and $\mathbf{E}^\ell$ is a set of tunable weights. In this paper, we chose ϕ^ℓ to be the linear rectifier, i.e., $\phi^\ell(v) = \max(0, v)$. The bottom layer activation was chosen to be the logistic link, i.e., $\phi^0(\mathbf{z}) = 1/(1 + exp(-\mathbf{z})$.

Note that in the RAE, the input to the decoder is now $\mathbf{z} = \hat{\mathbf{z}}^L$, i.e., the noise sample vector is set equal to top-most layer of neural activities of the encoder. The data log likelihood that the RAE optimizes is:

$$\psi = \sum_j \Big(\mathbf{x}[j] \log \mathbf{z}^0[j] + (1 - \mathbf{x}[j]) \log(1 - \mathbf{z}^0[j]) \Big) \tag{1}$$

where updates to each weight matrix $\mathbf{E}^\ell$ (of the encoder) and $\mathbf{W}^\ell$ (of the decoder) are updated by computing the relevant gradients $\frac{\partial \psi}{\partial \mathbf{E}}$ and $\frac{\partial \psi}{\partial \mathbf{W}}$, respectively. The weight gradients are then used to update model parameters via gradient ascent.

Gaussian Variational Auto-encoder (GVAE): Instead of using an encoder to only produce a single value for $\mathbf{z}$, we could instead modify this network to produce the parameters of a distribution over $\mathbf{z}$ instead. If we assume that this distribution is a multivariate Gaussian with a mean μ_z and a diagonal covariance $\sigma_z^2 = \mathbf{\Sigma}_z \odot \mathbf{I}$, we can then modify the RAE's encoder function to instead be: $(\mu_z, \sigma_z^2) = \text{NN}_e(\mathbf{x})$. Specifically, the top-most layer of $\text{NN}_e(\mathbf{x})$ is actually split into two separate output layers as follows: $\mu_z = \mathbf{E}^L_\mu \cdot \mathbf{z}^{L-1}$ and $\sigma_z^2 = \exp(\mathbf{E}^L_\sigma \cdot \mathbf{z}^{L-1})$ (this is also known as the variational autoencoder, or VAE [23]). $\mathbf{E}^L_\mu$ is the tunable weight matrix for the mean and $\mathbf{E}^L_\sigma$ is the tunable weight matrix for the variance. The data log likelihood for the GVAE is as follows:

$$\psi = \sum_j \Big(\mathbf{x}[j] \log \mathbf{z}^0[j] + (1 - \mathbf{x}[j]) \log(1 - \mathbf{z}^0[j])\Big) - \text{D}_{KL}\Big(q(\mathbf{z}|\mathbf{x}) || p(\mathbf{z})\Big) \quad (2)$$

where $q(\mathbf{z}|\mathbf{x}) = \mathcal{N}(\mu_z, \sigma_z^2)$ (the Gaussian parameters produced by the encoder $\text{NN}_e(\mathbf{x})$) and $p(\mathbf{z}) = \mathcal{N}(\mu_p, \sigma_p^2)$ where $\mu_p = 0$ and $\sigma_p^2 = 1$ (an assumed unit Gaussian prior over $\mathbf{z}$). The second term in the above objective is the Kullback-Leibler divergence D_{KL} between the distribution defined by the encoder and the assumed prior distribution. This term is meant to encourage the output distribution of the encoder to match a chosen prior distribution, acting as a powerful probabilistic regularizer over the model's latent space. Note that this divergence term serves as a top-down pressure on the top-most layer of the encoder while the gradients that flow from the encoder (via the chain rule of calculus) act as a sort of bottom-up pressure. Note that since $NN_e(\mathbf{x})$ is a distribution, the input to the decoder is, unlike the RAE, a sample of the encoder-controlled Gaussian, i.e., $\mathbf{z} = \mu_z + \sqrt{\sigma_z^2} \odot \epsilon$ where $\epsilon \sim \mathcal{N}(0, 1)$.

Gradients of the likelihood in Equation 2 are then taken with respect to all of the encoder and decoder parameters, including the new mean and variance encoder weights $\mathbf{E}^L_\mu$ and $\mathbf{E}^L_\sigma$, which are subsequently updated using gradient ascent. All the other activation functions of the GVAE are set to be the linear rectifier, except for the output function ϕ^0 of the decoder, which, like the RAE, is set to be the logistic sigmoid.

Constant-Variance Gaussian Variational Auto-encoder (GVAE-CV): This model [14] is identical to the GVAE except that the variance parameters σ_z^2 of the encoder are omitted and a fixed (non-learnable) value is chosen instead for the variance (meaning that the diagonal covariance is collapsed further to a single scalar). The exact value for this variance meta-parameter, for each benchmark, was chosen from the range $[0.025, 1.0]$ by tuning performance to a held-out set of image samples.

Generative Adversarial Network Autoencoder (GAN-AE): This model, also referred to as an adversarial autoencoder [29], largely adheres to the architecture of the GVAE except that the second term, i.e., the Kullback-Leibler divergence term, in the data log likelihood is replaced with the adversarial objective normally used to train implicit density estimators like the generative adversarial network (GAN) [15]. As a result, we integrate a third feedforward network, i.e., $p_r = \text{NN}_d(\mathbf{z})$, into the generative model (this module is also referred to as the discriminator). The discriminator is tasked with distinguishing whether an input vector comes from the desired prior distribution $p(\mathbf{z})$ (set to be a unit Gaussian as in the GVAE) or comes from the encoder network $\text{NN}_e(\mathbf{z})$ distribution. This task is posed as a binary classification problem, where a sample from the encoder $\mathbf{z}_f \sim \mathcal{N}(\mu_z, \sigma_z^2)$ is assigned the label of $c = 0$ (fake sample) and a sample drawn from the prior $\mathbf{z}_r \sim \mathcal{N}(0, 1)$ is assigned a label of $c = 1$ (real sample). These fake and real samples are fed through the discriminator which returns a scalar value for each, representing the probability $p_r = p(c = 1|\mathbf{z})$. This leads to the modified data log likelihood objective below:

$$\psi = \sum_j \Big(\mathbf{x}[j] \log \mathbf{z}^0[j] + (1 - \mathbf{x}[j]) \log(1 - \mathbf{z}^0[j])\Big) + \Big(\log(\text{NN}_d(\mathbf{z}_r)) + (1 - \log(\text{NN}_d(\mathbf{z}_f)))\Big). \quad (3)$$

However, to update the weights of the GAN-AE, we do not compute partial derivatives of Equation 3 directly. Instead, following in line with the typical multi-step optimization of [15, 29], upon presentation

of a sample or mini-batch of samples, we compute gradients with respect to $\text{NN}_e(\mathbf{x})$, $\text{NN}(\mathbf{z})$, and $\text{NN}_d(\mathbf{z})$ separately. Specifically, if we group all of the encoder weights under Θ_{NN_e}, all of the decoder weights under Θ_{NN}, and all of the discriminator weights Θ_{NN_d}, then the gradients of the objective are computed in three separate but successive steps shown below:

$$\Delta_{auto} = \frac{\partial \sum_j \Big(\mathbf{x}[j] \log \mathbf{z}^0[j] + (1 - \mathbf{x}[j]) \log(1 - \mathbf{z}^0[j])\Big)}{\partial(\Theta_{NN_e} \cup \Theta_{NN})} \quad \text{(Autoencoder gradients)} \quad (4)$$

$$\Delta_{gen} = \frac{\partial\Big(1 - \log(\text{NN}_d(\mathbf{z}_f))\Big)}{\partial \Theta_{NN}} \quad \text{(Generator gradients)} \quad (5)$$

$$\Delta_{disc} = \frac{\partial\Big(\log(\text{NN}_d(\mathbf{z}_r)) + (1 - \log(\text{NN}_d(\mathbf{z}_f)))\Big)}{\partial \Theta_{NN_d}} \quad \text{(Discriminator gradients)} \quad (6)$$

where the above gradient calculations are each followed by a separate gradient ascent update to their relevant target parameters, i.e., Δ_{auto} is used to update $\Theta_{NN_e} \cup \Theta_{NN}$, Δ_{gen} is used to update Θ_{NN_e}, and Δ_{disc} is used to update $\Theta_{NN_e} \cup \Theta_{NN_d}$.

The number of synaptic weights associated with the discriminator were included in the model's total parameter count and had two hidden layers of linear rectifier units. Again, like the GVAE, the hidden layer functions ϕ^ℓ of the encoder and decoder were chosen to be the linear rectifier and the output ϕ^0 of the decoder was set to be the logistic sigmoid.

Supplementary Note 8: On the Feature Analysis of Neural Generative Coding

The analysis we conducted (in Table 3 of the main paper) on the GNCN-t2-LΣ's intermediate representations involved, using the MNIST dataset, examining the generative synaptic weights for each layer of a trained GNCN-t2-LΣ model. Specifically, when we viewed the weight vectors that conveyed predictions of state neurons in layer 1 to layer 0 (the output), we found that the features resembled rough strokes and digit components (of different orientations/translations). When we viewed the weight vectors relating neurons in layer 2 to layer 1 and layer 3 to layer 2 , we found that they rather resembled neural selection "blueprints" or maps that seem to be used to select or trigger lower-level state neurons.

In Figure 6 of the main paper, we illustrate how these higher-level maps seem to interact with the low-level stroke visual features/dictionary. Based on our simple analysis, it appears that the GNCN-t2-LΣ learns a type of multi-level command structure, where neurons in one level learn to turn off and turn on neurons in the neighboring level below them, further scaling those they choose to activate by an intensity coefficient. When we reach layer 1, the state neurons chosen from the command structure of layers 2 and 3, as well as their final produced intensity coefficients (ranging from $[0, \infty)$ due to the fact that any layer of the GNCN-t2-LΣ in this paper uses the linear rectifier activation function) resemble and work to produce a composition of low-level features that ultimately produce a complete object or digit. This means that the GNCN-t2-LΣ (as well as the other predictive processing models like those of [13], i.e., the GNCN-t1-Σ, and [40], i.e., the GNCN-t1, since they process sensory inputs in the same way as the GNCN-t2-LΣ) learns to compose and produce a weighted summation of low-level features akin to the results of sparse coding [33] driven by a complex, higher-level neural latent structure. In the "Output" column of Table 3 of the main paper, we empirically confirm this by summing up the top most highly-activated state neurons in layer $\mathfrak{N}^1$ (multiplying each by its activation coefficient) – this simple super-position visually yields digits quite similar to the original one presented to the GNCN-t2-LΣ model.

While this simple feature analysis is promising, we remark that future work should involve developing methodology to map an NGC model's layerwise activities to those of actual brain activity (using fMRI data) or to a biological model such as HMAX, using methodology similar to that proposed in [39].

Supplementary References

[1] Ackley, D. H., Hinton, G. E., and Sejnowski, T. J. A learning algorithm for boltzmann machines. *Cognitive science 9*, 1 (1985), 147–169.

[2] Andersen, P., Eccles, J., and Løyning, Y. Hippocampus of the brain: Recurrent inhibition in the hippocampus with identification of the inhibitory cell and its synapses. *Nature 198*, 4880 (1963), 540–542.

[3] Baldi, P., Sadowski, P., and Lu, Z. Learning in the machine: Random backpropagation and the learning channel. *arXiv preprint arXiv:1612.02734* (2016).

[4] Bartunov, S., Santoro, A., Richards, B., Marris, L., Hinton, G. E., and Lillicrap, T. Assessing the scalability of biologically-motivated deep learning algorithms and architectures. In *Advances in Neural Information Processing Systems* (2018), pp. 9390–9400.

[5] Bengio, Y., Yao, L., Alain, G., and Vincent, P. Generalized denoising auto-encoders as generative models. In *Advances in neural information processing systems* (2013), pp. 899–907.

[6] Crafton, B., West, M., Basnet, P., Vogel, E., and Raychowdhury, A. Local learning in rram neural networks with sparse direct feedback alignment. In *2019 IEEE/ACM International Symposium on Low Power Electronics and Design (ISLPED)* (2019), IEEE, pp. 1–6.

[7] Crick, F. The recent excitement about neural networks. *Nature 337*, 6203 (1989), 129–132.

[8] Devlin, J., Chang, M.-W., Lee, K., and Toutanova, K. Bert: Pre-training of deep bidirectional transformers for language understanding. *arXiv preprint arXiv:1810.04805* (2018).

[9] Douglas, R. J., Koch, C., Mahowald, M., Martin, K., and Suarez, H. H. Recurrent excitation in neocortical circuits. *Science 269*, 5226 (1995), 981–985.

[10] Dumoulin, V., Goodfellow, I. J., Courville, A., and Bengio, Y. On the challenges of physical implementations of rbms. *arXiv preprint arXiv:1312.5258* (2013).

[11] El-Boustani, S., Ip, J. P., Breton-Provencher, V., Knott, G. W., Okuno, H., Bito, H., and Sur, M. Locally coordinated synaptic plasticity of visual cortex neurons in vivo. *Science 360*, 6395 (2018), 1349–1354.

[12] Frenkel, C., Lefebvre, M., and Bol, D. Learning without feedback: Direct random target projection as a feedback-alignment algorithm with layerwise feedforward training. *arXiv preprint arXiv:1909.01311* (2019).

[13] Friston, K. Hierarchical models in the brain. *PLoS computational biology 4*, 11 (2008), e1000211.

[14] Ghosh, P., Sajjadi, M. S., Vergari, A., Black, M., and Schölkopf, B. From variational to deterministic autoencoders. In *International Conference on Learning Representations* (2020).

[15] Goodfellow, I., Pouget-Abadie, J., Mirza, M., Xu, B., Warde-Farley, D., Ozair, S., Courville, A., and Bengio, Y. Generative adversarial nets. In *Advances in neural information processing systems* (2014), pp. 2672–2680.

[16] Hebb, D. O., et al. The organization of behavior, 1949.

[17] Hinton, G. E. Training products of experts by minimizing contrastive divergence. *Neural computation 14*, 8 (2002), 1771–1800.

[18] Hinton, G. E. To recognize shapes, first learn to generate images. *Progress in brain research 165* (2007), 535–547.

[19] Hinton, G. E., et al. What kind of graphical model is the brain? In *IJCAI* (2005), vol. 5, pp. 1765–1775.

[20] Hinton, G. E., and McClelland, J. L. Learning representations by recirculation. In *Neural information processing systems* (1988), pp. 358–366.

[21] Hopfield, J. J. Neural networks and physical systems with emergent collective computational abilities. *Proceedings of the national academy of sciences 79*, 8 (1982), 2554–2558.

[22] JADERBERG, M., CZARNECKI, W. M., OSINDERO, S., VINYALS, O., GRAVES, A., SILVER, D., AND KAVUKCUOGLU, K. Decoupled neural interfaces using synthetic gradients. In *Proceedings of the 34th International Conference on Machine Learning-Volume 70* (2017), JMLR. org, pp. 1627–1635.

[23] KINGMA, D. P., AND WELLING, M. Auto-encoding variational bayes. *arXiv preprint arXiv:1312.6114* (2013).

[24] LEE, D.-H., ZHANG, S., FISCHER, A., AND BENGIO, Y. Difference target propagation. In *Joint European Conference on Machine Learning and Knowledge Discovery in Databases* (2015), Springer, pp. 498–515.

[25] LILLICRAP, T. P., COWNDEN, D., TWEED, D. B., AND AKERMAN, C. J. Random synaptic feedback weights support error backpropagation for deep learning. *Nature communications 7* (2016), 13276.

[26] LITTLE, W. A. The existence of persistent states in the brain. *Mathematical biosciences 19*, 1-2 (1974), 101–120.

[27] LONDON, M., ROTH, A., BEEREN, L., HÄUSSER, M., AND LATHAM, P. E. Sensitivity to perturbations in vivo implies high noise and suggests rate coding in cortex. *Nature 466*, 7302 (2010), 123–127.

[28] LONG, N. M., LEE, H., AND KUHL, B. A. Hippocampal mismatch signals are modulated by the strength of neural predictions and their similarity to outcomes. *Journal of Neuroscience 36*, 50 (2016), 12677–12687.

[29] MAKHZANI, A., SHLENS, J., JAITLY, N., AND GOODFELLOW, I. Adversarial autoencoders. In *International Conference on Learning Representations* (2016).

[30] MOSTAFA, H., RAMESH, V., AND CAUWENBERGHS, G. Deep supervised learning using local errors. *Frontiers in neuroscience 12* (2018), 608.

[31] MOVELLAN, J. R. Contrastive hebbian learning in the continuous hopfield model. In *Connectionist Models*. Elsevier, 1991, pp. 10–17.

[32] NØKLAND, A. Direct feedback alignment provides learning in deep neural networks. In *Advances in Neural Information Processing Systems* (2016), pp. 1037–1045.

[33] OLSHAUSEN, B. A., AND FIELD, D. J. Emergence of simple-cell receptive field properties by learning a sparse code for natural images. *Nature 381*, 6583 (1996), 607–609.

[34] O'REILLY, R. C. Biologically plausible error-driven learning using local activation differences: The generalized recirculation algorithm. *Neural computation 8*, 5 (1996), 895–938.

[35] ORORBIA, A. G., HAFFNER, P., REITTER, D., AND GILES, C. L. Learning to adapt by minimizing discrepancy. *arXiv preprint arXiv:1711.11542* (2017).

[36] ORORBIA, A. G., AND MALI, A. Biologically motivated algorithms for propagating local target representations. In *Proceedings of the AAAI Conference on Artificial Intelligence* (2019), vol. 33, pp. 4651–4658.

[37] ORORBIA, A. G., MALI, A., KIFER, D., AND GILES, C. L. Deep credit assignment by aligning local representations. *arXiv preprint arXiv:1803.01834* (2018).

[38] PASCANU, R., MIKOLOV, T., AND BENGIO, Y. On the difficulty of training recurrent neural networks. In *International conference on machine learning* (2013), pp. 1310–1318.

[39] RAMAKRISHNAN, K., SCHOLTE, H. S., GROEN, I. I., SMEULDERS, A. W., AND GHEBREAB, S. Visual dictionaries as intermediate features in the human brain. *Frontiers in computational neuroscience 8* (2015), 168.

[40] RAO, R. P., AND BALLARD, D. H. Predictive coding in the visual cortex: a functional interpretation of some extra-classical receptive-field effects. *Nature neuroscience 2*, 1 (1999).

[41] RICHARDSON, E., AND WEISS, Y. On gans and gmms. *arXiv preprint arXiv:1805.12462* (2018).

[42] Santurkar, S., Schmidt, L., and Madry, A. A classification-based study of covariate shift in gan distributions. In *International Conference on Machine Learning* (2018), PMLR, pp. 4480–4489.

[43] Scellier, B., Goyal, A., Binas, J., Mesnard, T., and Bengio, Y. Generalization of equilibrium propagation to vector field dynamics. *arXiv preprint arXiv:1808.04873* (2018).

[44] Wacongne, C., Changeux, J.-P., and Dehaene, S. A neuronal model of predictive coding accounting for the mismatch negativity. *Journal of Neuroscience 32*, 11 (2012), 3665–3678.
